# Evidence Gaps and Global Patterns in Leishmaniasis Control: A Scoping Review of Clinical, Diagnostic, and Treatment Strategies

**DOI:** 10.1155/japr/8882865

**Published:** 2026-05-21

**Authors:** Muhi-deen Wonwana Mohammed, Yussif Owusu Aboagye, Akwasi Anyanful, Samuel Kyei, Kwame Kumi Asare

**Affiliations:** ^1^ Biomedical and Clinical Research Centre, College of Allied Health Sciences, University of Cape Coast, Cape Coast, Ghana, ucc.edu.gh; ^2^ Department of Medical Biochemistry, School of Medical Sciences, University of Cape Coast, Cape Coast, Ghana, ucc.edu.gh; ^3^ Department of Optometry, School of Allied Health Sciences, University of Cape Coast, Cape Coast, Ghana, ucc.edu.gh; ^4^ Department of Immunology, Noguchi Memorial Institute for Medical Research, University of Ghana, Accra, Ghana, ug.edu.gh

**Keywords:** cutaneous leishmaniasis, diagnosis, global burden, leishmaniasis, mucocutaneous leishmaniasis, neglected tropical diseases, relapse, scoping review, treatment, visceral leishmaniasis

## Abstract

**Background:**

Leishmaniasis is a neglected tropical disease with diverse clinical forms, heterogeneous geographical distribution, and persistent diagnostic and treatment challenges. Although substantial primary research exists, evidence remains fragmented, limiting integrated and context‐specific control strategies. This scoping review synthesizes global evidence on disease distribution, diagnostic performance, and treatment outcomes, and identifies critical evidence gaps.

**Methods:**

A scoping review was conducted in accordance with the Arksey and O′Malley framework and PRISMA‐ScR guidelines. Four electronic databases were searched for studies published between January 2000 and January 2026. Data were synthesized narratively. A total of 118 studies were included in the review, of which 75 were analyzed to assess the geographical distribution of Leishmaniasis, while the remaining 43 studies were used to evaluate diagnostic tools and treatment outcomes.

**Results:**

Seventy‐five studies from 53 countries were included. Cutaneous leishmaniasis was reported in 58 studies (77.3%) and was the most geographically widespread form, whereas visceral leishmaniasis was reported in 26 studies (34.7%) and showed marked focality, particularly in East Africa, South Asia, and Brazil. Molecular diagnostics demonstrated superior performance, with PCR and qPCR sensitivities ranging from 89% to 100% and specificities from 57% to 100%, depending on target gene and sample type. Loop‐mediated isothermal amplification showed sensitivities of 89.7%–95% and specificities of 63.6%–100%. In contrast, microscopy showed lower sensitivity (32.8%–80.8% for cutaneous and 44%–67% for visceral leishmaniasis) but high specificity (> 95%). Serological tests and rapid diagnostic tests performed well for visceral leishmaniasis, with rK39‐based assays showing sensitivities of 78–96.4% and specificities of 93%–97%, and direct agglutination tests achieving sensitivities of 97.3%–99.1% and specificities of 97.5%–98.8%. Fewer than one‐third of studies evaluated operational feasibility. Liposomal amphotericin B achieved the highest cure rates for visceral leishmaniasis, whereas antimonials remained the most frequently used treatment for cutaneous disease. Relapse was predominantly reported in visceral leishmaniasis.

**Conclusion:**

Leishmaniasis exhibits substantial global heterogeneity, with molecular diagnostics offering high analytical accuracy but limited real‐world deployment. Addressing operational constraints and integrating emerging technologies are critical priorities for strengthening global leishmaniasis control. Molecular and antigen‐based diagnostics offer improved accuracy but remain underutilized in resource‐limited settings. Effective treatment is complicated by toxicity, cost, and relapse. Expanding diagnostic capacity, optimizing treatment protocols, and strengthening surveillance systems are critical for effective global leishmaniasis control and elimination.

## 1. Introduction

Leishmaniasis is a complex group of parasitic diseases caused by protozoa of the genus *Leishmania*, transmitted to humans through the bite of infected female phlebotomine sandflies [1, 2]. The disease manifests in several clinical forms: cutaneous leishmaniasis (CL), visceral leishmaniasis (VL) (or kala‐azar), mucocutaneous leishmaniasis (MCL), mucosal leishmaniasis (ML), and post–kala‐azar dermal leishmaniasis (PKDL), each with distinct epidemiological patterns, clinical features, and public health implications [3, 4]. Globally, leishmaniasis affects 12 million people across 98 countries, with more than 1 billion individuals at risk, primarily in tropical and subtropical regions of Africa, South America, the Middle East, and Asia [5, 6]. According to the World Health Organization (WHO), an estimated 700,000–1 million new cases occur annually, with CL accounting for the majority, followed by VL and MCL [7].

Despite its global burden and impact on vulnerable populations, leishmaniasis remains a neglected tropical disease (NTD), often overlooked in health policy and underfunded in research [8, 9]. The disease predominantly affects low‐income populations, where delayed diagnosis, suboptimal treatment, poor access to healthcare, and limited surveillance exacerbate morbidity and mortality. The clinical diagnosis of leishmaniasis is particularly challenging due to its varied presentations, overlap with other infectious diseases, and the absence of a universal diagnostic algorithm [10–12]. In recent decades, the development of molecular diagnostics, serological assays, rapid diagnostic tests (RDTs), and antigen detection platforms has significantly improved diagnostic capabilities, although their implementation in endemic areas remains inconsistent [10, 11].

Treatment of leishmaniasis is equally complex and varies by disease form, *Leishmania* species, host factors, and geographical location [13–15]. Therapeutic options include pentavalent antimonials, liposomal amphotericin B (L‐AmB), miltefosine, paromomycin, and combination regimens, many of which are associated with toxicity, high costs, long durations, or limited efficacy against certain strains [16, 17]. Treatment failures, relapse, and the emergence of drug resistance further complicate disease management [18, 19]. Moreover, the rise in imported leishmaniasis cases in nonendemic countries due to global migration, travel, and climate change highlights the need for improved detection and reporting systems across all regions [20, 21].

Although numerous studies have examined different facets of leishmaniasis including epidemiology, diagnostics, and therapeutics, there remains a gap in synthesizing this knowledge to inform comprehensive control strategies. Scoping reviews provide an effective method to map existing evidence, identify research gaps, and guide policy and practice, particularly for multifaceted diseases such as leishmaniasis. This scoping review is aimed at systematically examining and summarizing current evidence on the geographical distribution, diagnostic tools, and treatment outcomes of leishmaniasis across various global contexts. By doing so, it seeks to inform public health interventions, strengthen surveillance, and support the development of effective, context‐specific approaches to control and eliminate this persistent NTD.

## 2. Methodology

### 2.1. Study Design

This scoping review was conducted to systematically map the existing literature on the geographical distribution, diagnostic accuracy, and treatment of leishmaniasis. The review followed the framework proposed by Arksey and O′Malley and enhanced by the Joanna Briggs Institute (JBI) guidelines for scoping review. This study employed a scoping review design, guided by the Preferred Reporting Items for Systematic Reviews and Meta‐Analyses extension for Scoping Reviews (PRISMA‐ScR) checklist [22–24]. A scoping review was chosen due to its utility in mapping a broad field of research, identifying key concepts, summarizing evidence, and highlighting research gaps particularly useful for diseases like leishmaniasis that present in diverse clinical forms and settings. The review is aimed at synthesizing current knowledge on the geographical distribution, diagnostic tools, and treatment outcomes of leishmaniasis globally (Tables S2, S3, and S4). This review protocol was registered with the Open Science Framework (OSF), hosted by the Center for Open Science (COS), Washington DC, under the registration link [25].

### 2.2. Objectives

The primary objective of this scoping review was to systematically collate and synthesize evidence on leishmaniasis with respect to three key domains: (1) the geographical distribution and proportional burden of its various clinical forms; (2) the diagnostic tools used and their reported accuracy; and (3) treatment modalities, outcomes, relapse rates, and adverse effects. This synthesis was intended to inform policy, improve diagnostic and treatment strategies, and guide future research efforts in global leishmaniasis control.

### 2.3. Eligibility Criteria

Studies were included if they met specific inclusion criteria: They had to be published between January 2000 and January 2026, report on human cases of leishmaniasis, and focus on at least one of the review′s three domains: epidemiology, diagnostics, or treatment. Only peer‐reviewed primary research articles and relevant grey literature with adequate methodological detail were included. Studies could be based in either endemic or nonendemic regions. Exclusion criteria encompassed non‐English publications, review articles, editorials, conference abstracts without full text, case reports with fewer than five patients, and studies focused solely on animal models or vector biology without direct clinical or public health relevance.

### 2.4. Information Sources and Search Strategy

A comprehensive search strategy was employed to identify eligible studies. Searches were conducted in four major databases: Google Scholar, PubMed, Scopus, and Science Direct. Additional articles were identified through backward and forward citation tracking from reference list screening of included full‐text articles. Search terms included a combination of Medical Subject Headings (MeSH) and free‐text keywords such as “leishmaniasis,” “cutaneous leishmaniasis,” “visceral leishmaniasis,” “mucocutaneous leishmaniasis,” “diagnosis,” “treatment,” “epidemiology,” and “relapse.” Boolean operators and truncation were used to maximize retrieval. The search was tailored to the requirements of each database and restricted to studies published between 2000 and 2026. A total of 118 studies were included in the review, of which 75 were analyzed to assess the geographical distribution of Leishmaniasis, while the remaining 43 studies were included in the data use in evaluate diagnostic tools and treatment outcomes (Table S1).

### 2.5. Study‐Selection Process

**Figure 1:**
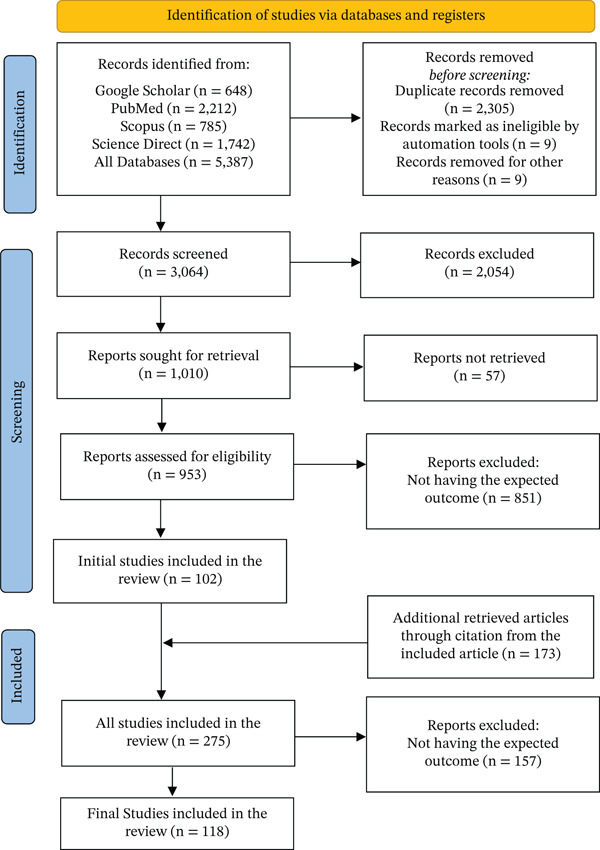
PRISMA flowchart of search and selection of the included studies.

All retrieved citations were imported into Zotero for reference management and duplicate removal. Titles and abstracts were independently screened by two reviewers to assess eligibility. Full‐text articles were then reviewed in detail to confirm inclusion based on the predefined criteria. Disagreements were resolved through discussion or arbitration by a third reviewer. The entire selection process was documented using the PRISMA flow diagram, which details the number of records identified, screened, excluded, and included in the final synthesis (Figure 1).

### 2.6. Data Extraction and Charting

Data were extracted using a standardized and piloted data extraction form. Two reviewers independently extracted information on study title, authors, year of publication, country of study, study design and duration, setting (endemic or nonendemic), population characteristics, type and form of leishmaniasis, diagnostic methods and their performance (sensitivity, specificity), treatment regimens, clinical outcomes, adverse events, and reported relapse rates. Extracted data were cross‐verified for consistency, and discrepancies were resolved by consensus.

### 2.7. Data Synthesis and Analysis

The extracted data were synthesized thematically, aligning with the three central domains of the review: geographical distribution, diagnostics, and treatment outcomes. A narrative synthesis approach was used to summarize findings across studies. Descriptive statistics such as frequencies and proportions were calculated to illustrate trends in disease burden, diagnostic accuracy, and treatment response. Tables and figures were used to facilitate comparison and visualization, including country‐specific burden, diagnostic performance metrics, and therapeutic efficacy across regions.

### 2.8. Quality Considerations

In line with the purpose of a scoping review, no formal risk‐of‐bias assessment or quality scoring was undertaken. However, the methodological clarity, sample size, and relevance of each study were critically considered during data interpretation. Studies that lacked essential methodological detail or had incomplete outcome reporting were noted but not excluded if they contributed meaningful information to the overall synthesis (Tables S3 and S4).

## 3. Results

### 3.1. Geographical Distribution and Proportional Burden of Different Forms of Leishmaniasis

The distribution of leishmaniasis varied widely by country, clinical form, and population studied. VL was the predominant clinical form reported in several countries, including Brazil, where 69/111 (62.2%) VL cases were identified following the exclusion of other forms of *Leishmania* infection [26]. In addition to VL, Brazil bears a substantial burden of American tegumentary leishmaniasis (ATL), predominantly comprising CL (1292/1470; 87.9%), followed by MCL (174/1470; 11.8%), and a small proportion of other unidentified *Leishmania* forms (4/1470; 0.3%), which together contribute significantly to the clinical spectrum of leishmaniasis in the country [27], as well as in Ethiopia [28–30], India [31, 32], China [33–35], Tunisia [36], Pakistan [37], Kuwait [38], Somalia [39], Honduras [40], Bolivia [41], Qatar [42], Venezuela [43], Chad [44], Niger [45], Georgia [46, 47], Thailand [48], and Palestine [49], where VL accounted for over 90% of reported cases. In contrast, CL dominated in countries across North Africa, the Middle East, and parts of South America, reflecting its global prevalence. High‐burden countries reporting exclusively or nearly exclusively CL included Algeria [50] (~217,741 cases), Afghanistan [51] (~148,945), Iran [52–54], Morocco [55–57], Turkey [58], and Saudi Arabia [59–63]. Other countries with smaller burdens but predominantly CL included Jordan [64], Bangladesh [65], Libya [66], Kenya [67, 68], Montenegro [69], Ghana [70], the United States [71], Armenia [72], France [73], Malta [74], Suriname [75], the United Kingdom [76], Peru [77], Germany [78], Guinea [79], Bulgaria [80], Colombia [81], Panama [82], Spain [83, 84], Senegal [85], Mauritania [86], Israel [87], Italy [88], and Lebanon [89], each reporting more than 90%–95% of cases as cutaneous. TL was rare and geographically limited, appearing primarily in Argentina [90], Brazil [27, 91], and Turkmenistan [92]. Across studies, the variation in leishmaniasis forms reflected both endemicity and population characteristics, with VL concentrated in hotspot regions and CL widely distributed. The patterns align with global WHO observations, showing that CL is the most common clinical form, VL is focal yet severe, and TL remains uncommon worldwide (Figure 2).

Figure 2Global distribution of leishmaniasis forms. (a). Leishmaniasis type distribution across included countries in the study, (b). distribution of leishmaniasis forms by included countries with less than 2000 reported positive cases, (c). distribution of leishmaniasis forms by included countries with more than 2000 positive cases. The figure was generated using Microsoft Corporation Power BI, online software, 2026.

**Figure:**
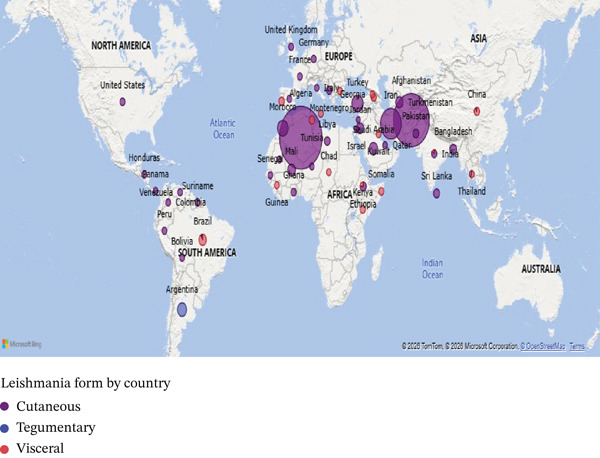
(a)

**Figure:**
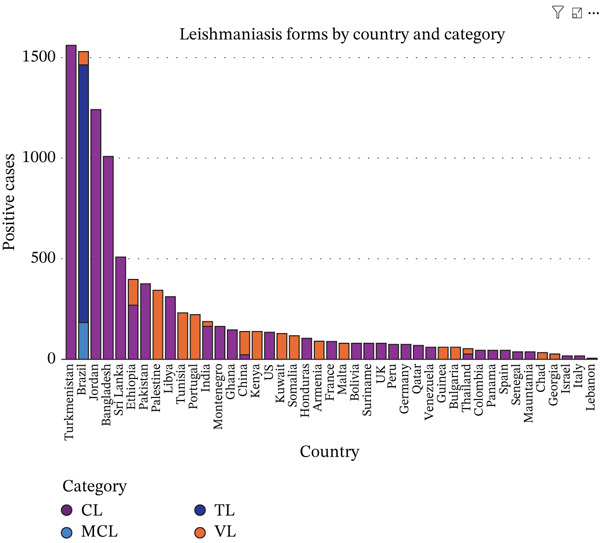
(b)

**Figure:**
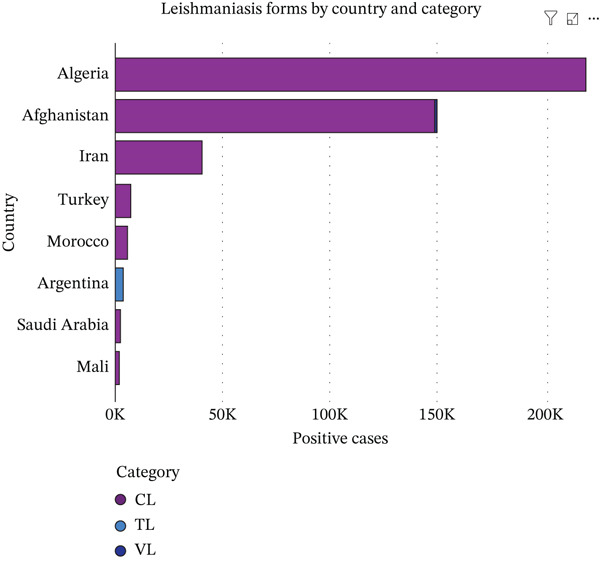
(c)

### 3.2. Global Distribution of Leishmaniasis: Evidence from Multinational Observational Studies

The evidence from 75 studies published between 2000 and 2025, encompassing data from 53 countries or country‐regions worldwide [26–100]. The included studies span all major geographical regions affected by leishmaniasis: Africa, the Middle East, Asia, Europe, and the Americas, providing a comprehensive overview of the global distribution and epidemiological patterns of the disease [26–100].

The methodological approaches were heterogeneous. Retrospective observational studies (*n* = 33) [26, 27, 29, 31, 33, 34, 36, 39, 40, 43, 44, 49–51, 54–58, 61–63, 68–70, 72–78, 80, 82, 84–92, 94, 96, 97, 100] and cross‐sectional surveys (*n* = 18) [30, 37, 38, 42, 44, 45, 47, 48, 52, 60, 63, 65, 70, 79, 95, 98, 99] constituted the majority of the evidence base, followed by descriptive observational studies (*n* = 11) [27, 49, 58, 61, 64, 67, 83, 91, 93, 96, 99]. Fewer studies employed cohort designs (*n* = 5) [28, 40, 45, 65, 96], prospective or longitudinal approaches (*n* = 4) [44, 46, 53, 80], or interventional trial designs (*n* = 4) [32, 41, 71, 81]. Study durations ranged from short‐term investigations of 4 months [30] to long‐term surveillance extending up to 69 years [69].

Of the 75 studies, 55 (73.3%) were conducted in endemic settings [26–36, 39–41, 43, 44, 46, 47, 49–51, 53, 56, 58–64, 68–74, 76, 77, 79, 80, 82, 84–93, 95–97, 99, 100], 12 (16.0%) originated from nonendemic countries [35, 42, 55, 71, 73, 76, 78, 80, 83, 84], and 8 (10.7%) did not explicitly report endemicity status [45, 48, 52, 57, 67, 94, 96, 98]. Studies from endemic countries contributed the largest cumulative case numbers and frequently consisted of long‐term retrospective or surveillance datasets [50, 51, 53, 58, 69, 97]. In contrast, studies from nonendemic countries including Germany, France, the United Kingdom, Qatar, Bulgaria, Malta, and the United States primarily documented imported cases among travelers, migrants, military personnel, or immunocompromised individuals, often reporting very high or complete positivity among clinically suspected cohorts [35, 42, 71, 73, 74, 76, 78, 80, 83, 84].

The populations investigated were highly heterogeneous and included general community populations [40, 53, 56, 58, 60, 64, 70, 95, 98], healthcare‐facility attendees [29, 30, 33, 50, 52, 59, 61–63, 90], dermatology and infectious disease patients [36, 49, 83, 85], children [54, 74], people living with HIV [48], nomadic pastoralists [68], migrants and travelers [35, 73, 76, 78, 80, 83, 92], and military personnel [77]. Community‐based surveys frequently demonstrated substantial disease presence in general populations [53, 58, 60, 70, 95], whereas hospital‐ and referral‐based studies often reported very high detection rates among symptomatic individuals, in some cases reaching 100% positivity in narrowly defined clinical subgroups [39, 42, 52, 55, 61, 76, 78, 80, 83].

Regarding clinical presentation, CL was the most frequently reported form, documented in 58 studies (77.3%) [26–28, 30,32, 34, 35, 37, 40–45, 47, 49–53, 55–66, 68–71, 77, 78, 82–87, 89, 92, 95–100], underscoring its dominance across diverse epidemiological settings. VL was reported in 26 studies (34.7%), with marked geographic clustering in East Africa, South Asia, the Middle East, and parts of South America [26, 29, 31, 33, 36, 38, 39, 46, 48, 51, 54, 65, 67, 68, 72, 74, 76, 80, 84, 86–88, 90, 93, 94]. MCL was documented in 11 studies (14.7%), largely from Latin America, with occasional imported cases reported in Europe [27, 41, 42, 51, 71, 73, 76, 84, 88]. TL was the least frequently reported form, identified in seven studies (9.3%), predominantly from Brazil and neighboring regions [27, 51, 90, 91].

The numerical distribution of studies and countries [26–100], highlights the extensive geographical reach and marked heterogeneity of leishmaniasis worldwide. The findings emphasize the predominance of endemic‐region data, the substantial contribution of long‐term surveillance studies to global burden estimates, and the consistent dominance of CL, alongside geographically restricted but clinically severe forms such as visceral and MCL (Table 1 and Figure 2).

**Table 1:** The geographical distribution and proportional burden of the various clinical leishmaniasis forms.

Author (reference)	Year	Country/Region	Study design	Population type	Study Duration	Positive cases *n*/*N*	Endemic/nonendemic	Form of leishmaniasis			
								CL	VL	MCL	TL
Dabirzadeh et al. [52]	2024	Iran	Cross‐sectional	Healthcare facility	2018–2020 (2 years)	32/32	Not stated	x			
Abukhattab et al. [42]	2024	Qatar	Retrospective, cross‐sectional	General population	2016–2022 (5 years)	68/68	Nonendemic	x	x	x	
Alharbi and Ahmed [59]	2024	Saudi Arabia	Retrospective, observational	Healthcare facility	2020–2022 (2 years)	2280/2280	Endemic	x			
Yizengaw et al. [28]	2024	Ethiopia	Cohort study ^∗^	CL patients presenting to a treatment center	2019–2022 (3 years)	264/346	Endemic	x			
Lindner et al. [78]	2024	Germany	Retrospective chart review	Travelers, migrants	2000–2023 (3 years)	75/75	Nonendemic	x			
Rocha et al. [93]	2024	Portugal	Retrospective study	Patients diagnosed in public hospitals; immunosuppressed groups	2010–2020 (10 years)	221/221	Endemic		x		
Debash et al. [29]	2023	Ethiopia	Retrospective study	Healthcare facility	2017–2021 (4 years)	132/564	Endemic		x		
Alzahrani et al. [60]	2023	Saudi Arabia	Community‐based cross‐sectional survey	General population	January to October 2022 (9 months)	149/391	Endemic	x			
Aflatoonian et al. [53]	2022	Iran	Longitudinal study	General population	1971–2020 (49 years)	40,164/40,164	Endemic	x			
Abdullahi et al. [67]	2022	Kenya	Descriptive cross‐sectional and retrospective study	Community‐based	2010–2020 (10 years)	78/360	Not stated		x		
Aissaoui et al. [73]	2021	France	Retrospective review	Travelers; immunocompromised	2012–2020 (8 years)	89/89	Nonendemic	x	x		
Aalto et al. [39]	2020	Somalia	Retrospective review ^∗^	Symptomatic patients with suspected VL	2013–2019 (6 years)	118/118	Endemic		x		
Alhawarat et al. [64]	2020	Jordan	Retrospective study	General population	2010–2016 (6 years)	1243/1243	Endemic	x			
Kanyina [68]	2020	Kenya	Retrospective review	Nomadic pastoralists	May–October 2014 (6 months)	136/136	Endemic		x		
Bisetegn et al. [30]	2020	Ethiopia	Cross‐sectional study	Healthcare facility	February to May 2019 (4 months)	46/205	Endemic	x			
Baghad et al. [55]	2020	Morocco	Cross‐sectional study	Patients with CL	2010–2016 (6 years)	106/106	Nonendemic	x			
Abou‐Elaaz et al. [56]	2019	Morocco	Not specified	General population	1998–2015 (17 years)	5518/5518	Endemic	x			
Hawash et al. [61]	2018	Saudi Arabia	Descriptive study	Healthcare facility	2016–2017 (1 year)	90/90	Endemic	x			
Mokhtar et al. [94]	2017	Saudi Arabia	Not specified	Healthcare facility		32/384	Not stated		x		
Castro et al. [81]	2017	Colombia	Open‐label trial	General population	2012–2013 (1 year)	46/60	Not stated	x			
Elmekki et al. [62]	2017	Saudi Arabia	Cross‐sectional study	Healthcare facility	2012–2015 (3 years)	467/467	Endemic	x			
Helel et al. [36]	2017	Tunisia	Retrospective (case‐control)	CL patients presenting to a treatment center	2004–2014 (10 years)	230/230	Endemic		x		
Traoré et al. [95]	2016	Mali	Cross‐sectional study	General population	2014 (1 year)	548/1428	Endemic	x			
El Aasri et al. [57]	2016	Morocco	Retrospective study	Affected population in rural and urban areas	2006–2014 (8 years)	415/415	Not stated	x			
Abdinia et al. [54]	2016	Iran	Retrospective study	Children	2000–2015 (15 years)	156/156	Endemic		x		
Araujo et al. [26]	2016	Brazil	Retrospective study	General population	2007–2013 (6 years)	69/69	Endemic		x		
Vita et al. [27]	2016	Brazil	Descriptive study	General population	2004–2013 (9 years)	1470/1470	Endemic	x		x	x
Oré et al. [77]	2015	Peru	Descriptive study ^∗^	Military personnel	2010 (8 months)	76/303	Endemic	x			
Calderaro et al. [88]	2014	Italy	Retrospective, observational study	Healthcare facility	1992–2013 (21 years)	15/134	Endemic	x	x		
Yemisen et al. [58]	2012	Turkey	Descriptive study	General population	2001–2008 (7 years)	7172/7172	Endemic	x			
Oliveira et al. [96]	2009	Mali	Cohort study	General population	2006–2008 (2 years)	1530/1530	Not stated	x			
Alsamarai and Alobaidi [97]	2009	Iraq	Prospective survey	Patients presenting at dermatology clinic	October 2004–April 2005 (7 months)	107/107	Endemic	x			
Negera et al. [98]	2008	Ethiopia	House‐to‐house survey	General population	2005–2006 (1 year)	92/1907	Not stated	x			
Vieira‐Gonçalves et al. [91]	2008	Brazil	Retrospective, descriptive study	General population	1991–2006 (15 years)	71/71	Endemic				x
Lawn et al. [76]	2004	United Kingdom	Retrospective, observational study	Travelers	1995–2003 (8 years)	79/79	Nonendemic	x		x	
Al‐Qurashi et al. [63]	2000	Saudi Arabia	Retrospective, descriptive, and comparative study	Healthcare facility	1994–1996 (2 years)	16/73	Endemic	x			
Israël et al. [44]	2024	Chad	Prospective cross‐Sectional Survey	Healthcare facility	2019–2020 (1 year)	33/40	Endemic		x		
Vutova et al. [80]	2024	Bulgaria	Prospective survey	Healthcare facility and travelers	1976–2021 (45years)	58/58	Endemic		x		
Saadene et al. [50]	2023	Algeria	Retrospective	Healthcare facility	2000–2020 (20years)	217,741/217,741	Endemic	x			
Almazán et al. [90]	2023	Argentina	Retrospective	Healthcare facility	1985–2019 (34years)	3573/3573	Endemic				x
Chowdhury et al. [65]	2019	Bangladesh	Exposed and nonexposed cohort	Healthcare facility	2004–2008 (4years)	1011/1011	Endemic	x			
Zhao et al. [33]	2025	China	Retrospective	Healthcare facility	2014–2023 (9 years)	89/89	Endemic		x		
Kuhls et al. [72]	2021	Armenia	Not stated	Healthcare facility	2012–2016 (4 years)	91/91	Endemic		x		
Ballart et al. [41]	2021	Bolivia	Not stated	Healthcare facility	2014–2015	80/135	Endemic	x		x	
Adegboye O.A. and Adegboye M. [51]	2017	Afghanistan	Retrospective	Healthcare facility	2003–N (6 years)	148,945/148,945	Endemic	x	x	x	
Diadie et al. [85]	2018	Senegal	Retrospective observational	Dermatology patients	2010–2014	38/38	Endemic	x			
Garrido‐Jareño et al. [83]	2020	Spain	Observational and retrospective study	Patients	2006–2017	42/42		x		x	
Galgamuwa et al. [99]	2018	Sri Lanka	Not stated	Patients	2009–2016	8487	Endemic	x			
Iddawela et al. [100]	2018	Sri Lanka	Case series	Patients	2005–2015	509/509	Endemic	x			
Díaz‐Sáez et al. [84]	2022	Spain	Prospective study	Patients	2014–2016	24/24	Endemic	x	x	x	
Schallig et al. [75]	2019	Suriname	Not stated	Patients	2016–2017	79/93	Endemic	x			
Larréché et al. [92]	2013	Turkmenistan		Travelers	2000–2009	1562	Endemic	x			
Zhao et al. [34]	2021	China	Case series	Inpatients	2008–2018	111/111	Endemic		x		
Bi et al. [35]	2024	China	Not stated	Migrants (patients)	2015–2022	25/72	Nonendemic	x			
De Lima et al. [43]	2009	Venezuela	Not stated	Patients	1992–1995	63/63	Endemic	x			
Jundang et al. [48]	2024	Thailand	Cross‐sectional study	HIV patients	2020–2021	56/650	Not stated	x	x		
Ahmad [49]	2020	Palestine	Descriptive epidemiological study	Patients	1990–2017	343/343	Endemic		x		
Pinart et al. [71]	2020	United States	Randomized controlled trials	Patients	7 years	134/134	Endemic	x			
Medenica et al. [69]	2023	Motenegro	Retrospective	People affected by leishmaniosis (or leishmaniasis)	1945–2014 (69 years)	165/165	Endemic	x			
Iqbal W. et al. [37]	2024	Pakistan	Cross‐sectional	Leishmaniosis patients	2019–2020	374/374	Endemic	x			
Iqbal J. et al. [38]	2002	Kuwait	Cross‐sectional	VL patients, suspected VL patients, and healthy individual as control	Not stated	130/323	Endemic		x		
Grech et al. [74]	2000	Malta	Retrospective study	Pediatric patients with VL	1980–1998 (18 years)	81/81	Endemic		x		
Gonzalez et al. [82]	2020	Panama	Not stated	Patients with positive laboratory and clinical diagnoses of LCL	January–December 2012	46/46	Endemic	x			
El Moctar et al. [86]	2025	Mauritania	Cross‐sectional descriptive study	Compatible skin lesions	2022–2024 (2 years)	37/37	Endemic	x			
El Hajj et al. [89]	2018	Lebanon	Retrospective	Patients with suspected VL	2014–2017 (3 years)	5/5	Not stated		x		
Blaizot et al. [45]	2025	Niger	Prospective cohort	Patients with clinically suspected CL lesions	2023–2024 (1 year)	33/59	Not stated	x			
Amro et al. [66]	2017	Libya	Not stated	CL patients	2011–2012 (1 year)	312/312	Endemic	x			
Solomon et al. [87]	2019	Israel	Retrospective	Suspected CL patients	1993–2015 (22 years)	17/145	Endemic	x		x	
Akuffo et al. [70]	2021	Oti Region (Ghana)	Cross‐sectional	Residents (≥ 12 months, aged between 2–65 years)	2018–2021 (3 years)	150/595	Endemic	x			
Kumar et al. [31]	2020	Bihar (Vaishali), India	Retrospective analysis	Generally rural	2015–2016	26/622,849	Endemic		x		
Kato et al. [46]	2016	Georgia (Kakheti)	Epidemiological survey	Local inhabitants	2014–2015	25/33	Endemic		x		
Babuadze et al. [47]	2016	Georgia (Kakheti)	Cross‐sectional sero‐survey	General (1–70 years)	2014–2015	39/513	Endemic		x		
Kimutai et al. [79]	2017	Guinea/West Africa	Systematic review	General population	12 years	60/3126	Endemic		x		
Sosa‐Ochoa et al. [40]	2025	Honduras (Amapala)	Cohort study	General population	24 months	104/576	Endemic	x			
Thakur et al. [32]	2022	India (Himachal Pradesh)	Systemic‐immune profiling	General population	24 months	161 total	Endemic	x			

### 3.3. Diagnostic Accuracy of Tools for Leishmaniasis: A Multistudy Analysis of Molecular, Serological, and Microscopic Approaches

The included studies employed a diverse set of diagnostic tools to detect various forms of leishmaniasis, including CL, VL, MCL, PKDL, oral leishmaniasis, ATL, and nonulcerated cutaneous leishmaniasis (NUCL) [26, 27, 31–38, 40–52, 54, 57, 58, 61–66, 69–72, 74–77, 79–92, 94–130]. The most frequently reported causative species were Leishmania donovani, Leishmania infantum, Leishmania major, Leishmania tropica, Leishmania braziliensis, Leishmania guyanensis, Leishmania panamensis, Leishmania lainsoni, and Leishmania mexicana. Diagnostic approaches included polymerase chain reaction (PCR) and its variants, loop‐mediated isothermal amplification (LAMP), microscopy, culture, serological assays (ELISA, IFAT, and IHA), direct agglutination tests (DAT), RDTs, and skin‐based tests such as the leishmanin skin test (LST) and Montenegro skin test (MST).

PCR and qPCR were the most widely employed molecular tools. Dabirzadeh et al. [52] reported 93.75% sensitivity for CL, whereas Adams et al. [105] demonstrated qPCR sensitivity of 98% and specificity of 84% for CL. Bensoussan et al. [118] showed ITS1‐PCR achieved 91% sensitivity and 100% specificity, whereas kDNA‐PCR showed 98.7% sensitivity but lower specificity (57.1%). Hossain et al. [127] reported 100% sensitivity for real‐time PCR in VL, 85% in PKDL, and variable performance in relapsed cases. Noninvasive swab PCR methods, evaluated by Boni et al. [122], demonstrated sensitivities of 92.6% (kDNA PCR) with 80% specificity, highlighting potential for field‐adaptable diagnostics.

Microscopy remained a common diagnostic method, particularly in resource‐limited settings, but with variable sensitivity. For CL, sensitivity ranged from 32.8% to 80.8% [121, 125], whereas for VL, it ranged from 44% to 67% [124, 127]. Culture methods, used alongside microscopy, generally had lower sensitivities (32.8%–62.8%) but high specificity [121, 124]. Histopathology and immunohistochemistry were applied in MCL and oral leishmaniasis, offering direct visualization but limited sensitivity in early‐stage infections [115, 116].

Serological tests and antigen detection were particularly effective for VL. Abeijon et al. [104] reported urine‐based ELISA sensitivities of 91.66% (Brazil) and 93.33% (Kenya), both with 100% specificity. Salotra et al. [130] found rk39 ELISA sensitivity of 94.5% and specificity of 93.7%, whereas amastigote and promastigote ELISAs ranged from 86.4% to 92% sensitivity. IFAT and EIA assays also performed well; Skraba et al. [129] reported 93.3% sensitivity and 90.8% specificity, and Grech et al. [74] reported IFAT sensitivity of 97.5% (95% CI: 90.5%–99.6%).

DAT proved highly reliable for VL. Ayelign et al. [113] reported AQ‐DAT sensitivity and specificity of 97.3% and 98.8%, respectively, and FD‐DAT sensitivity of 99.1% with specificity of 97.5%. rK39‐based RDTs were widely used in the field, with sensitivities ranging from 78% to 96.4% and specificities of 93%–97% [35, 113, 117].

LAMP assays showed high accuracy with simpler protocols. Adams et al. [108] reported sensitivities of 95% for CL and 92% for VL, with specificities of 86%–100%. Aerts et al. [109] reported LAMP sensitivity of 89.7% and specificity of 63.6% for CL, highlighting suitability for point‐of‐care applications.

LST and MST were used in CL and ATL epidemiological studies. Boggild et al. [121] reported MST sensitivity ranging from 60.7% to 95.8% and specificity up to 83.3%, whereas Oliveira et al. [96] effectively applied LST for community‐level mapping.

Sensitivity and specificity often varied by *Leishmania* species and sample type. For instance, qPCR for *L. guyanensis* and related species in the Amazon region achieved 68%–73% sensitivity and 97% specificity, whereas microscopy sensitivities were lower (51%–76%) but specificity remained near 99% [120].

From the evidence, molecular techniques demonstrated superior sensitivity, especially for early‐stage and nonulcerative infections, whereas serological assays and RDTs were highly effective for VL. Traditional methods such as microscopy and culture, although less sensitive, remained critical where molecular tools were unavailable. LAMP and noninvasive PCR‐based diagnostics are emerging as promising tools for field‐based detection and surveillance (Table 2).

**Table 2:** Leismaniasis diagnostic methods used and their reported accuracy.

Author (s)	Form of Leishmaniasis	Causative parasite (s)	Diagnostic methods	Reported sensitivity/specificity										
				PCR		Microscopy/culture		ELISA	EIA	DAT	LAMP	RDT	IFAT	MST/LST
Mokh et al. [94]	VL	Leishmania donovani.	Indirect hemagglutination assay (IHAT).	—		—		—	—	—	—	—	—	—
Castro et al. [81]	CL	Leishmania panamensis, Leishmania braziliensis.	PCR.	—		—		—	—	—	—	—	—	—
Dabirzadeh et al. [52]	CL	Leishmania major and Leishmania tropica.	Microscopy, PCR, and culture.	Sensitivity: 93.75%.		—		—	—	—	—	—	—	—
Hawash et al. [61]	CL	Leishmania amastigote.	Microscopy and PCR.	Sensitivity: 100%.		Sensitivity: 74%.		—	—	—	—	—	—	—
Abukhattab et al. [42]	CL (88.23%), VL (10.29%), mucocutaneous leishmaniasis (MCL) (1.47%) 9	Not stated.	Not stated.	—		—		—	—	—	—	—	—	—
Gorski et al. [101]	VL	L. donovani.	DAT (immunological). Microscopy. rK39 RDT (rapid diagnostic test).	—		—		—	—	—	—	—	—	—
Oliveira et al. [96]	CL	L. major.	Leishmanin skin test (LST)	—		—		—	—	—	—	—	—	—
Traoré et al. [95]	CL	L. major.	LST, PCR, and antisaliva antibodies by ELISA.	—		—		—	—	—	—	—	—	—
Negera et al. [98]	CL	Leishmania aethiopica.	Culture, histopathology, and PCR.	—		—		—	—	—	—	—	—	—
Rajni et al. [102]	CL	L. tropica.	Microscopy, histopathologic examination, antigen testing, and PCR.	—		—		—	—	—	—	—	—	—
Abbasi et al. [103]	VL	L. donovani and L. major.	PCR.	—		—		—	—	—	—	—	—	—
Abeijon et al. [104]	VL	Leishmania infantum and L. donovani.	Urine‐based antigen detection assay (ELISA).	—		—		Sensitivity: 91.66% (Brazil), 93.33% (Kenya) Specificity: 100%	—	—	—	—	—	—
Adams et al. [105]	CL	Not stated.	Microscopy, qPCR, and culture.	qPCR: sensitivity: 98% (95% CI: 91%–100%) specificity: 84% (95% CI: 64%–95%).		—		—	—	—	—	—	—	—
Adams et al. [106]	VL	L. donovani.	Direct agglutination test (DAT).	—		—		—	—	—	—	—	—	—
Abdalla et al. [107]	CL	L. major.	Microscopy, LST, and PCR.	—		—		—	—	—	—	—	—	—
Adams et al. [108]	VL and CL	L. donovani, L. infantum (VL) and L. tropica, L. major, L. braziliensis, Leishmania mexicana, L. panamensis, and L. guyanensis (CL).	LAMP, microscopy, culture, and qPCR.	—		—		—	—	—	CL: sensitivity: 95% (95% CI, 87.2%–98.5%), specificity: 86% (95% CI, 67.3%–95.9%). VL: sensitivity: 92% (95% CI, 74.9%–99.1%), specificity 100% (95% CI, 85.8%–100%)	—	—	—
Aerts et al. [109]	CL	L. major and L. tropica.	Microscopy, LAMP, RDT, and PCR.	—		Sensitivity: 78.97%, specificity: 77.27%.		—	—	—	Sensitivity: 89.68% specificity: 63.64%	Sensitivity: 66.27% specificity: 95.45%	—	—
Aghamolaei et al. [110]	CL	L. major.	Microscopy and kDNA‐nested‐PCR6.	—		—		—	—	—	—	—	—	—
Alborzi et al. [111]	MCL	L. major.	Microscopy and PCR (nested PCR).	—		—		—	—	—	—	—	—	—
Alabaz et al. [112]	VL	L. infantum (40%), L. donovani (26.7%), and L. tropica (23.3%) 8.	Microscopy, RDT (rK39 dipstick test), PCR (genus specific PCR, real‐time PCR, and ITS1 PCR‐RFLP), and DNA sequencing.	—		—		—	—	—	—	—	—	—
Ayelign et al. [113]	VL	L. donovani.	Microscopy, DAT (AQ‐DAT and FD‐DAT), rK39‐ICT (RDT).	—		—		—	—	AQ‐DAT: sensitivity: 97.3% (95% CI: 93.7–98.7)/specificity 98.8% (95% CI: 96.4–99.8) FD‐DAT: sensitivity: 99.1% (95% CI: 95.6–100)/specificity 97.5% (95% CI: 95.2–98.1)	—	rK39‐ICT: sensitivity: 96.4% (95% CI: 91.8–98.7)/specificity: 93.2% (95% CI: 90.1–94.8)	—	—
Almeida et al. [114]	Oral leishmaniasis	Not stated.	Microscopy and immunohistochemistry.	—		—		—	—	—	—	—	—	—
Azizi et al. [115]	Oral leishmaniasis	Not stated.	Histology (microscopic examination of biopsy) and bone marrow examination.	—		—		—	—	—	—	—	—	—
Barçante et al. [116]	American tegumentary leishmaniasis (ATL)	Leishmania spp.	Histopathology, imprint with staining (microscopy), and PCR	—		—		—	—	—	—	—	—	—
Bangert et al. [117]	VL	L. donovani and L. infantum.	RDT (rK39‐ICT), agglutination (DAT), immunofluorescence (IFAT). For gold standard: PCR, microscopy, and culture.	—		—		—	—	Sensitivity: 86.5% (80.5–92.5)	—	rK39‐ICT: sensitivity: 78.0% (70.8–85.2)	Sensitivity: 79.4% (72.4–86.4)	—
Bensoussan et al. [118]	CL	L. major, L. tropica, L. braziliensis.	PCR, microscopy, culture, and serology.	ITS1 PCR: sensitivity: 91.0%, specificity: 100%. kDNA PCR: sensitivity: 98.7%, specificity: 57.1%. SLME PCR: sensitivity: 53.8%, specificity: 100%.		Microscopy: sensitivity: 74.4%, specificity: 100%. Culture: sensitivity: 62.8%, specificity: 100%.		—	—	—	—	—	—	—
Beyhan et al. [119]	CL	L. tropica.	Microscopy, culture, PCR (ITS‐1), and sequencing.	—		—		—	—	—	—	—	—	—
Bezemer et al. [120]	CL	Leishmania guyanensis, L. braziliensis, L. lainsoni, and L. naiffi.	qPCR and microscopy.	Amazon region: qPCR: sensitivity: 68% (95%CrI 49%–82%), specificity: 97% (95%CrI 93%–99%) Pacific region: qPCR: sensitivity: 73% (95%CrI 63%–83%), specificity: 97% (95%CrI 93%–99%).		Amazon region: sensitivity: 51% (95%CrI 36%–66%), specificity: 99% (95%CrI 96%–100%) Pacific region: sensitivity: 76% (95%CrI 65%–86%), specificity 99% (95%CrI 96%–100%).		—	—	—	—	—	—	—
Boggild et al. [121]	CL	Leishmania (V.) braziliensis, L. (V.) peruviana, L. (V.) guyanensis, L. (V.) lainsoni, and L. (V.) braziliensis/L. (V.) peruviana hybrid.	PCR (kDNA PCR, PCR‐RFLP), microscopy (Giemsa‐stained smears), culture, and LST.	kDNA PCR of invasive specimens: sensitivity: 92.5% (95% CI 86.2–98.8), specificity: 88.9% (95% CI 74.4–100), kDNA PCR of lesion aspirates: sensitivity: 80.6% (95% CI 71.1–90.1), specificity: 94.4% (95% CI 83.8–100), kDNA PCR of lesion scrapings: sensitivity: 79.1% (95% CI 69.4–88.8), specificity: 94.4% (95% CI 83.3–100), kDNA PCR of noninvasive specimens (FPLI): sensitivity: 86.6% (95% CI 78.4–94.8), specificity: 88.9% (95% CI 74.4–100).		Microscopy: sensitivity: 49.3% (95% CI 37.3–61.3), specificity: 100.0% culture: sensitivity: 32.8% (95% CI 21.6–44), specificity: 100.0%.		—	—	—	—	—	—	LST: sensitivity: 60.7% (95% CI 49–72.4), specificity: 83.3% (95% CI 66.1–100)
Boni et al. [122]	ATL, including cutaneous leishmaniasis (CL) and mucosal leishmaniasis (ML)	Leishmania braziliensis.	Swab samples (noninvasive) with PCR‐kDNA, PCR‐HSP70, and qPCR‐HSP70. Compared against biopsy samples with PCR‐kDNA (considered the gold standard for this study).	G36PCR‐kDNA on swab: sensitivity: 92.6%, specificity: 80%. PCR‐HSP70 on swab: sensitivity: 40.7%, specificity: 100%. qPCR: sensitivity: 33.3%.		—		—	—	—	—	—	—	—
Brustoloni et al. [123]	VL	Not stated.	Microscopy, culture, and serology (IFAT).	—		BMA culture: sensitivity: 60.5%.		—	—	—	—	—	Sensitivity: 86.1%	—
Cruz et al. [124]	VL	L. donovani and L. infantum.	Serology (IFAT, ELISA, and dipstick), antigen detection (latex agglutination), microscopy, culture, and PCR (LnPCR, snPCR‐RFLP).	LnPCR (peripheral blood): sensitivity: 79% LnPCR (bone marrow): sensitivity: 100%.		Microscopy: sensitivity: 67%, culture: sensitivity: 44%.		Ranged from 89% to 96%	—	—	—	Katex: sensitivity: 69%	—	—
Conter et al. [125]	CL	Leishmania (Viannia) braziliensis.	Microscopy (DS), serology (IIF), skin test (MST), and PCR (kDNA PCR, *β*‐globin PCR).	—		Sensitivity: 80.8%.		—	—	—	—	IIF: sensivity: 100%	—	MST Sensitivity: 95.8%.
Mahdavi et al. [126]	VL and CL	L. donovani complex, L. infantum 49. Also L. major, L. tropica, and L. brasiliensis.	ELISA, LFT, RDT, microscopy, and agglutination test.	—		—		—	—	—	—	Sensitivity: range from 91.2% to 97.1%, specificity: 93.6%–99.2%	—	—
Hossain et al. [127]	Visceral leishmaniasis (VL), relapsed visceral leishmaniasis (RVL), post kala‐azar dermal leishmaniasis (PKDL), cured VL, cured PKDL	L. donovani.	PCR (real‐time PCR, nested PCR), RDT, microscopy, agglutination test.	Real‐time PCR sensitiviy for VL and RVL: 100% (95% CI, 91.19%–100%) Real‐time PCR sensitivity for PKDL samples: 85.0% (95% CI, 70.16%–94.29%) Ln‐PCR sensitivity for VL samples: 77.5% (95% CI, 61.55%–89.16%), Ln‐PCR sensitivity for RVL samples: 100% (95% CI, 69.15%–100%), Ln‐PCR sensitivity for PKDL samples: 52.5% (95% CI, 36.1368.49%).		—		—	—	—	—	—	—	—
Prestes et al. [128]	ML	L. (V.) braziliensisand Leishmania (Viannia) guyanensis.	PCR (conventional, RFLP), and microscopy.	—		—		—	—	—	—	—	—	—
Skraba et al. [129]	American cutaneous leishmaniasis (ACL)	L. (V.) braziliensis.	EIA (enzyme immunoassay), SDS‐PAGE, and western blotting.	—		—		—	Sensitivity: 93.3%, Specificity: 90.8%	—	—	—	—	—
Salotra et al. [130]	PKDL, VL/kala‐azar (KA)	L. donovani.	ELISA, PCR, and microscopy (direct parasitological diagnosis).	—		—		rk39 ELISA: sensitivity: 94.5%, specificity: 93.7%. Amastigote Antigen ELISA: Sensitivity: 92%. Specificity: 90.3%, promastigote antigen ELISA: sensitivity: 86.36%. Specificity: 90.2%	—	—	—	—	—	—
Israël et al. [44]	VL	L. donovani.	Serology (1 mL of the patient′s buffy coat to which two drops of formaldehyde), and PCR.	—		—		—	—	—	—	—	—	—
Vutova et al. [80]	VL	Not stated.	Serological methods, such as the indirect immunofluorescence assay test (IFAT), enzyme‐linked immunosorbent assay (ELISA), and polymerase chain reaction (PCR).	—		—		—	—	—	—	—	—	—
Almazán et al. [90]	TL	L. (V.) braziliensis.	Smear and/or culture and/or LST.	—		—		—	—	—	—	—	—	—
Kuhls et al. [72]	VL	L. donovani and L. infantum.	Microscopy and PCR.	—		—		—	—	—	—	—	—	—
Ballart et al. [41]	CL and MCL	L. (V.) braziliensis.	Microscopy and culture.	—		—		—	—	—	—	—	—	—
Diadie et al. [85]	CL	Leishmania spp.	Microscopy.	—		—		—	—	—	—	—	—	—
Garrido‐Jareño et al. [83]	CL/MCL	L. infantum.	Microscopy.	—		—								
Iddawela et al. [100]	CL	L. donovani.	Microscopy.	Sensitivity approaching 100%.		High specificity and low sensitivity.								
Pinart et al. [71]	CL/MCL	L. mexicana. L. braziliensis, and L. panamensis.												
Díaz‐Sáez et al. [84]	VL/CL/MCL	L. infantum.	Microscopy, in vitro culture, PCR, urine antigen detection, and serology.											
Schallig et al. [75]	CL	L. (V.) guyanensis.	Microscopy.	CL‐detect sensitivity: 35.8%, specificity: 83.3%.	Loopamp sensitivity: 91.4%, specificity: 91.7%.	CL‐detected sensitivity: 36.7%, specificity: 85.7%	Loopamp sensitivity: 84.8%, specificity: 42.9%			**—**	**—**	**—**	**—**	**—**
Larréché et al. [92]	CL	L. major.	PCR and microscopy.											
Bi et al. [35]	VL	Not stated.	rK39 RDTs.	—		—		—	—	**—**	**—**	97%	**—**	—
De Lima et al. [43]	CL	L. infantum.	LST, PCR, and parasite isolation.											
Jundang et al. [48]	CL/VL	L. orientalis, L. martini quensis, and L. donovani.	PCR and DAT.											
Alhawarat et al. [64]	CL	Nonendemic parasites trains (L. major and L. donovani).	Positive smear or culture.											
Ahmad [49]	VL	L. infantum.	Microscopic examination.											
Solomon et al. [87]	CL and ML.	L. (V.) braziliensis.	PCR.	—		—		—	—	—	—	—	—	—
Amro et al. [66]	CL	L. tropica and L. major.	PCR and microscopy	—		—		—	—	—	—	—	—	—
Blaizot et al. [45]	CL	L. major.	Parasitological smear (microscop) and PCR.	—		—		—	—	—	—	—	—	—
El Hajj et al. [89]	VL	L. infantum.	PCR.	—		—		—	—	—	—	—	—	—
El Moctar et al. [86]	CL	L. major.	PCR and microscopy.	—		—		—	—	—	—	—	—	—
Gonzalez et al. [82]	CL	Leishmania (Viannia) panamensis.	Histopathology and PCR.	—		—		—	—	—	—	—	—	—
Grech et al. [74]	VL	L. donovan.i	Immunofluorescent antibody testing (IFAT).	—		—		—	—	—	—	—	Sensitivity: IFAT was 97.5% (95% CI: 90.5 to 99.6%).	—
Iqbal et al. [38]	VL	L. donovani and L. tropica	IHA, IFA, and strip‐test for IgG antibodies to leishmania antigen.	—		—		—	—	—	—	Sensitivity: 80% Specificity: 100%.	Sensitivity:86.6%, specificity:93%.	—
Iqbal et al. [37]	CL	L. amastigote.	Microscopy.	—		—		—	—	—	—	—	—	—
Medenica et al. [69]	CL, and VL	Not stated.	Parasitology and serology.	—		—		—	—	—	—	—	—	—
Akuffo et al. [70]	Cutaneous (CL)	Leishmania spp.	PCR and microscopy.	—		—		—	—	—	—	—	—	—
Kumar et al. [31]	Visceral (VL)	L. donovani.	rK39 RDT and microscopy.	—		—		—	—	—	—	—	—	—
Kato et al. [46]	Cutaneous (CL)	L. lainsoni.	PCR, smear, and culture.	—		—		—	—	—	—	—	—	—
Babuadze et al. [47]	Visceral (VL)	L. infantum.	PCR, rK39 and microscopy.	—		—		—	—	—	—	—	—	—
Kimutai et al. [79]	Visceral (VL)	L. donovani.	Microscopy (bone marrow).	—		—		—	—	—	—	—	—	—
Sosa‐Ochoa et al. [40]	Nonulcerated cutaneous (NUCL)	L. infantum.	ELISA and LST (DTH).	—		—		—	—	—	—	—	—	—
Thakur et al. [32]	Cutaneous (CL)	L. donovani.	ELIZA rK39 RDT.	—		—		—	—	—	—	—	—	—

*Note:* “—”: not applicable, “not stated”: result or the method was not reported, “not performed”: The method was not performed.

Abbreviations: CL, cutaneous leishmania; ELISA, enzyme‐linked immunosorbent assay; IFAT, immunofluorescence assay test; KA, kala‐azar leishmaniasis; MCL, mucocutaneous leishmania; ML, mucosal leishmania; NUCL, nonulcerated cutaneous leishmania; PKDL, post–kala‐azar dermal leishmaniasis; PCR, polymerase chain reaction;VL, visceral leishmania.

### 3.4. Treatment Outcomes and Adverse Effects of Leishmaniasis Therapies

The reviewed studies reported a wide range of pharmacological interventions for CL, VL, MCL, NUCL, and ML [26, 27, 31–38, 40–51, 53, 54, 57, 58, 61–63, 65, 66, 69–72, 74–77, 79–92, 94–143]. The most commonly used drugs were meglumine antimoniate (MA) (glucantime), sodium stibogluconate (SSG), amphotericin B formulations (liposomal, deoxycholate, or lipid complex), miltefosine, pentamidine, and fluconazole.

For CL, MA (either intramuscular or intralesional) was frequently employed, often in combination with cryotherapy, resulting in cure rates ranging from 62% to 91% [135, 136, 139]. SSG, delivered intralesionally or intramuscularly, also showed efficacy, with reports of partial or complete lesion resolution in most patients [32, 37, 61, 66]. Miltefosine, administered orally or intravenously, cured the majority of CL patients but was associated with gastrointestinal side effects including nausea and vomiting [71, 81]. Nonantimonial treatments such as fluconazole and metronidazole achieved high clinical cure rates (up to 95%) in some cohorts [45, 85]. Relapse was generally uncommon for CL, though some studies reported treatment failures or minor recurrences [133, 137].

VL treatment primarily involved L‐AmB, SSG, MA, paromomycin, and combination therapies [31, 44, 72, 131, 132, 138, 140]. Single‐dose L‐AmB achieved high efficacy with minimal adverse events and no reported relapses [31]. Combination therapies, such as SSG plus paromomycin or L‐AmB plus SSG, demonstrated cure rates of 73%–98% [101, 134, 138], though adverse effects like diarrhea, vomiting, injection site reactions, and rare fatalities were reported [74, 80, 131]. Relapse rates varied, with studies noting 0.7%–23% relapse in VL patients depending on treatment type and follow‐up duration [47, 131, 138].

Treatments for MCL and ML included amphotericin B, antimonial drugs, itraconazole, and pentamidine, often achieving high cure rates (81%–99%) but occasionally associated with relapse and adverse events including fever, chills, and acute kidney injury [42, 137, 143].

MA administered intramuscularly achieved 100% NUCL cure in treated cohorts, with only mild injection site pain and malaise reported, and no relapses observed [40].

Across all forms of leishmaniasis, antimonial therapies were associated with common side effects including injection site pain, malaise, arthralgias, and mild gastrointestinal symptoms [38, 40, 47, 81]. Amphotericin B formulations had relatively fewer adverse effects when given in liposomal form, but conventional deoxycholate formulations could cause fever, nephrotoxicity, or electrolyte imbalances [131, 143]. Combination regimens generally improved efficacy but increased the risk of side effects.

Relapse was most frequently reported in VL patients, particularly those receiving monotherapy or delayed treatment [131, 138, 140]. CL, MCL, and NUCL generally had low relapse rates, though isolated recurrences occurred in some cohorts [133, 137].

Thus, antimonials and amphotericin B remain the mainstays of treatment, with miltefosine emerging as an effective oral alternative. Combination therapies increase cure rates, whereas liposomal formulations minimize toxicity. Relapse monitoring remains essential, particularly in VL‐endemic regions (Table 3).

**Table 3:** Leishmaniasis treatment modalities, outcomes, relapse rates, and adverse effects.

Author (references)	Form of leishmaniasis	Treatment used	Route of administration	Treatment outcome	Treatment side effects	Relapse reported
Aflatoonian et al. [53]	CL	Meglumine antimoniate (MA)	Intramuscular (IM) and intralesional (IL)	Not stated.	Not stated.	Not reported.
Castro et al. [81]	CL	Miltefosine (Impavido)	IV	Nausea, vomiting, abdominal pain, and mildly increased creatinine levels.	Fifty‐two patients were cured, five pediatric patients failed treatment, and three participants were lost to follow‐up.	No.
Hawash et al. [61]	CL	Sodium stibogluconate (SSG)	IM	Not stated.	Forty patients (85.1%) displayed clinical signs for partial or complete resolution of their skin lesions.	No.
Abukhattab et al. [42]	CL, VL, and MCL	Amphotericin B (AmphoB) and Pentostam	Local/topical, IV, IL, and oral	Not stated.	Cured without sequelae (22.06%), cured with sequelae (10.29%), improved (2.94%). No mortality reported among those completing follow‐up.	No.
Gorski et al. [101]	VL	SSG/paromomycin (PM) (combination therapy), SSG (monotherapy)	SSG: IM. PM sulfate: IM injections	Diarrhea, bleeding, and vomiting.	Relapse and death during VL treatment.	Yes.
Lucero et al. [131]	VL	L‐AMB (AmBisome)	Intravenous (IV) infusion.	Serious adverse events (SAE) recorded for seven patients (0.5%). Other complications included bleeding (3.4%), vomiting (9.5%), and other (0.4% including fever, diarrheas, abdominal pain, local rash at injection site).	Cure rates at 6 months were 98.7%. Cure rates at 12 months were 96.4%.	Yes, 39 patients were diagnosed with relapse.
Romero et al. [132]	VL	(i) MA (standard treatment), (ii) (AmphoB) deoxycholate, (iii) Liposomal Amphotericin B (L‐AMB), and (iv) L‐AMB plus MA (combination).	IV for all treatments.	Not stated.	Per intention‐to‐treat (ITT) analysis (at 6 months): MA: 77.5% cure rate. L‐AMB: 87.2% cure rate. L‐AMB plus MA: 83.9% cure rate. Per‐protocol (PP) analysis (at 6 months): MA: 94.5% cure rate. LAMB: 92.2% cure rate. LAMB plus MA: 98.9% cure rate.	Yes, relapse was evaluated as a secondary endpoint, with nine cases observed in 315 patients.
Silva et al. [133]	CL	SSG.	IL.	Not stated.	Treatment failure: 75.1% (151/201 patients). Complete healing: 24.9% (50/201 patients).	Yes.
Verrest et al. [134]	VL	L − AMB + SSG; L − AMB + miltefosine; miltefosine monotherapy (conventional dose); Miltefosine monotherapy (allometric dose); Fexinidazole.	IV (L‐AMB), IM (SSG), Oral (miltefosine and fexinidazole).	Not stated.	High blood parasite loads on Day 28 and Day 56 after treatment are an early indication of VL relapse. Fexinidazole (Fexi10D) showed poor efficacy, with only 10% achieving adequate parasite clearance, and delayed immune response. AmB + SSG10D, AmB + MF10D, MFC28D, MFA28D showed higher efficacy, with 73%–86% achieving adequate parasite clearance by Day 56. Miltefosine monotherapy had a slower onset of clearance but achieved complete clearance by Day 56 due to long half‐life.	Yes.
Aflatoonian et al. [135]	CL	1. MA (Glucantime) + cryotherapy. 2. MA (Glucantime).	1. IL. 2. IM.	Not stated.	Overall treatment failure rate: 11% (combined treatments), 1. IL MA + cryotherapy: 91% cure rate. 9% failure rate. 2. IM MA alone: 81.4% cure rate. 18.5% failure rate.	Yes.
Ahmad et al. [136]	CL	1. MA (glucantime) 2. Hydroxychloroquine (plaquenil).	1. Systemic 2. Oral.	Not stated.	The lesion was completely healed at the end of the month of hydroxychloroquine treatment.	Yes.
Brilhante et al. [137]	CL, MCL	N‐methylglucamine antimony and AmphoB.	Not mentioned.	Not stated.	99.2% recorded as cured and 0.4% gave up treatment.	Yes (recurrences were 3.6% for cutaneous and 3.5% for mucous form).
Mbui et al. [138]	VL	1. SSG + PM (combination therapy); 2. Ambisome.	PM: IM. Ambisome: Intravenously.	Not stated.	98.6% initially cured, 0.5% were confirmed nonresponse, 0.2% was a probable nonresponse.	Yes, 5 (0.7%) relapses were diagnosed10. Low follow‐up rates (2.5%) hinder the detection of Post‐kala‐azar dermal leishmaniasis (PKDL) cases, which are a complication/relapse1112.
Abdellahi et al. [139]	CL	Group A: glucantime plus 50% trichloroacetic acid (TCA). Group B: IL glucantime. Group C: systemic glucantime.	A and B:IL. C: Systemic.	Not stated.	Cure rates: Group A: 80%. Group B: 62%. Group C: 42%.	No.
Burza et al. [140]	VL	L‐AMB (Ambisome).	IV.	Not stated.	Cure rate (99.3%).	Yes.
Ramesh et al. [141]	VL	1. SSG alone 2. (SSG + allopurinol − Year 1), (SSG + rifampicin = Year 2), (SSG + allopurinol + rifampicin − Year 3), (SSG + Mw vaccine − Year 4).	1. SSG: IV. 2. Allopurinol: Oral, 3. Rifampicin: Oral. 4. Mw vaccine: intradermal.	Bodyaches, giddiness, metallic taste, loss of appetite, severe joint pains (arthralgias), thrombophlebitis (cord‐like structures replacing veins), vomiting, and febrile episodes.	Cure rate for SSG only was 12.5% (4/32), SSG + allopurinol : 30% (3/10), SSG + rifampicin: 33.3% (3/6), SSG + rifampicin + allopurinol: 0% (0/5), SSG + Mw vaccine 100% (2/2).	No.
Farooq et al. [142]	CL	1. MA. 2. Chloroquine	1.MA: IM. 2. Chloroquine: Oral	Not stated.	MA showed better efficacy than chloroquine	Not reported.
Santos et al. [143]	ML	1. L‐AMB, 2. amphotericin B lipid complex (ABLC), 3. deoxycholate amphotericin B (d‐AMB), 4. itraconazole, 5. antimonial pentavalent, and 6. pentamidine.	Not mentioned.	Fever, chills, sweating, palpitations, phlebitis, nausea/vomiting, electrolyte imbalance, acute kidney injury.	The healing percentages were 81.3% for L‐AMB, d‐AMB (13.3%), ABLC (46.1%), antimonial pentavalent (56.0%), itraconazole (40.0%), and pentamine (72.7%)	Yes. Relapse rate of 7.7% in patients who were treated with a high rate of permanent interruption.
Israël et al. [44]	VL	MA (Glucantime).	Not mentioned.	Not mentioned.	No	No.
Vutova et al. [80]	VL	Glucantime.	Not stated.	Fatal outcome was observed in two patients (3.5%) during treatment, who developed acute respiratory and cardiovascular failure.	Success with the first therapeutic course in the majority of patients (79.3%).	Yes, relapses were observed in seven patients (12.1%).
Zhao et al. [33]	VL	SSG.	IV.	Not stated.	Out of 89 children treated, 65 were discharged.	No.
Kuhls et al. [72]	VL	MA (glucantime).	Not mentioned.	Not stated.	No.	Not stated.
Ballart et al. [41]	CL & MCL	Glucantime (MA) and AmphoB.	Not stated.	Fourteen patients were considered as treatment failure.	The large majority of patients receiving a second treatment completed it in the past (14/20; 70%) and, from those, 42.9% (n = 6) showed complete recovery for the past leishmaniasis episode.	Not stated.
Diadie et al. [85]	CL	Fluconazole.	Oral.	95% clinical cure.	Not stated.	No.
Garrido‐Jareño et al. [83]	CL/MCL	L‐AMB.	IL.	—	Mucosal involvement.	No reported relapse.
Pinart et al. [71]	CL/MCL	MA and oral miltefosine.	Oral.	Complete cure.	Myalgias and arthralgias.	—
Larréché et al. [92]	CL	Pentamidine base.	IM.	Effective.		
Bi et al. [35]	CL	SSG.	IL.	All recovered.		No relapse.
Ahmad et al. [49]	VL	Antimonial SSG (Pentostam1) solution, and iposomal AmphoB after two cases.				Two cases relapsed but cured after treatment.
Solomon et al. [87]	CL and ML	PM ointment, itraconazole, IL SSG, IV SSG, L‐AMB, and miltefosine.	Topical Oral IV IV IV.	Not stated.	The rest of the patients achieved cure.	Yes, relapse occurred in three patients in the L‐AmB group.
Amro et al. [66]	CL	SSG.	IM.	Not stated.	Not stated.	Not stated.
Blaizot et al. [45]	CL	Metronidazole.	Oral.	Not stated.	30/33 had a complete response.	Not stated.
El Hajj et al. [89]	VL	Abelcet.	IV.	Not stated.	All the five patients died.	No.
El Moctar et al. [86]	CL	Not stated.	Not stated.	Not stated.	Not stated.	Not stated.
Gonzalez et al. [82]	CL	Glucantime.	IM.	Not stated.	Not stated.	Not stated.
Grech et al. [74]	VL	SSG.	IV.	Not stated.	Derangement of liver function tests, microscopic haematuria, proteinuria, thrombocytopenia, diabetic ketoacidosis.	Relapse of VL occurred in nine of 60 patients.
Iqbal J. et al. [38]	VL	Not stated.	Not stated.	Not stated.	Not stated.	Not stated.
Iqbal W. et al. [37]	CL	SSG.	IL or IV.	Not stated.	During IM injections ulcerative lesions was observed for healing. The lesions resolution signs included size regression (Complete or partial scarring and re‐epithelialization) and reduction in inflammatory signs (skin edema, erythema, and hardening).	Not stated.
Medenica et al. [69]	CL and VL	Not stated.	Not stated.	Not stated.	Not stated.	Not stated.
Akuffo et al. [70]	CL	No specific clinical drug (observation).	N/A.	Self‐healing observed in many cases.	Not stated.	Not reported.
Kumar et al. [31]	VL	Single‐dose L‐AmB (AmBisome).	IV.	High efficacy.	Minor infusion reactions.	No relapse.
Kato et al. [46]	CL	Glucantime.	IM.	Complete healing.	Not stated.	Not stated.
Babuadze et al. [47]	VL	MA.	IM.	94% initial cure rate.	Injection site pain, malaise.	12 cases (23%).
Kimutai et al. [79]	VL	SSG and PM.	IV and IM.	Variable, often late presentation.	Injection site, injection site swelling, pyrexia, vomiting and abdominal pain.	Not reported.
Sosa‐Ochoa et al. [40]	NUCL	MA.	IM.	Benign evolution 100% cure in treated group.	Injection site pain and malaise.	0% during cohort.

*Note:* “—”not applicable, “not stated” result or the method was not reported, “not performed” the method was not performed.

Abbreviations: CL, cutaneous leishmania; IM, intramuscular; IV, intravenous; MA, meglumine antimoniate; MCL, mucocutaneous leishmania; ML, mucosal leishmania; NUCL, nonulcerated cutaneous leishmania; SSG, sodium stibogluconate; VL, visceral leishmania.

## 4. Discussion

This scoping review provides a comprehensive synthesis of global evidence on the epidemiology, diagnostic approaches, and treatment outcomes of leishmaniasis from 118 studies, highlighting the profound heterogeneity of the disease across geographical regions, clinical forms, and healthcare contexts. Drawing on data from 75 studies conducted in 53 countries, the findings reaffirm leishmaniasis as a persistent NTD with uneven advances in detection, management, and surveillance despite decades of research and control efforts [1–9].

The results confirm that CL remains the most prevalent and geographically widespread form of the disease, accounting for over three‐quarters of the included studies. This pattern mirrors WHO reports identifying CL as the dominant clinical form globally, particularly in North Africa, the Middle East, Central Asia, and Latin America [5–7]. In contrast, VL, although less frequent overall, demonstrated intense focality, with overwhelming predominance in East Africa, South Asia, and Brazil, where it accounted for more than 90% of reported cases in several endemic countries [27–31, 51].

These marked geographical differences reflect variation in *Leishmania* species distribution, sandfly vectors, reservoir hosts, climatic conditions, and socioeconomic determinants of health [3, 4, 144]. TL and MCL, though relatively rare, were largely confined to Latin America and associated with significant morbidity, supporting prior evidence that these forms contribute disproportionately to disability‐adjusted life years despite their lower incidence [27, 41, 91]. The detection of predominantly CL cases in nonendemic countries further emphasizes the growing relevance of imported leishmaniasis linked to migration, military deployment, travel, and climate‐driven vector expansion [20, 21, 73, 76, 78].

The review demonstrates substantial progress in leishmaniasis diagnostics, particularly with the adoption of molecular tools. PCR‐based methods, including qPCR and kDNA‐ or ITS‐targeted assays, consistently showed superior sensitivity and specificity compared with conventional microscopy and culture, especially for CL and early or non‐ulcerative infections [52, 105, 118, 122, 127]. These findings are consistent with previous reports that molecular diagnostics outperform parasitological methods, which are limited by low sensitivity, operator dependence, and reduced yield in chronic or pauciparasitic disease [10–12, 121, 125].

Serological assays and antigen‐based tests, notably rK39 ELISA and RDTs, as well as DAT, showed high diagnostic accuracy for VL and remain essential in endemic, resource‐limited settings [74, 104, 113, 117, 130]. However, their reduced sensitivity in HIV‐coinfected patients, relapse cases, and PKDL has been well documented and was also evident across several included studies [48, 127]. Skin‐based tests such as the Montenegro and LSTs demonstrated value for epidemiological mapping but limited utility for clinical diagnosis due to variable sensitivity and inability to distinguish active from past infection [96, 121].

The findings highlight the absence of a single universally optimal diagnostic test for leishmaniasis. Instead, they support the need for integrated diagnostic algorithms combining clinical assessment, serological screening, and confirmatory molecular testing, tailored to disease form, endemicity, and available infrastructure [10, 11, 113]. The limited implementation of high‐performance molecular diagnostics in many endemic regions underscores ongoing inequities in access to diagnostic innovation.

Although diagnostic accuracy is a critical determinant of test performance, an exclusive focus on sensitivity and specificity provides an incomplete picture of the real‐world utility of leishmaniasis diagnostics [145, 146]. In endemic, resource‐limited settings where the disease burden is highest operational characteristics such as sample requirements, infrastructure, cost, technical expertise, and turnaround time often determine whether a diagnostic tool can be effectively deployed [147–149]. Many of the highly sensitive PCR‐based assays require invasive or technically demanding samples (e.g., tissue aspirates or bone marrow), reliable electricity, advanced laboratory infrastructure, cold‐chain storage, and trained personnel, all of which limit their scalability and routine use at peripheral health facilities [150, 151]. Consequently, despite their superior analytical performance, PCR assays are often restricted to reference laboratories and research settings, reducing their impact on early case detection and timely treatment.

LAMP represents a partial advance toward field‐adaptable molecular diagnostics, as it requires simpler equipment, shorter processing times, and can be performed at a constant temperature [150]. However, the reviewed literature rarely evaluated LAMP beyond laboratory accuracy metrics, with limited assessment of its robustness under field conditions, reagent stability in tropical climates, or integration into existing diagnostic workflows [152]. Similarly, although serological tests and RDTs demonstrated lower sensitivity than molecular methods in some contexts, their minimal infrastructure requirements, rapid turnaround time, and ease of use make them more practical for frontline screening in endemic areas [150, 153]. The lack of standardized reporting on operational feasibility across studies limits direct comparison of diagnostic tools and underscores the need for future research to move beyond accuracy metrics toward implementation‐focused evaluations. Incorporating operational performance data is essential for informing evidence‐based diagnostic algorithms that balance accuracy with feasibility, sustainability, and equity in leishmaniasis‐endemic settings.

Consistent with global treatment guidelines, antimonial compounds and amphotericin B formulations remain the mainstay of therapy across most clinical forms of leishmaniasis [13–17]. For CL, MA and SSG achieved moderate to high cure rates, particularly when administered intralesionally or combined with cryotherapy, with generally low relapse rates [32, 37, 61, 66, 135, 136]. Oral miltefosine emerged as an effective alternative, offering operational advantages, although gastrointestinal toxicity and concerns about resistance and teratogenicity persist [71, 81].

For VL, L‐AmB consistently demonstrated the highest efficacy and most favorable safety profile, corroborating WHO recommendations for its use as first‐line therapy where available [16, 17, 31]. Combination therapies, including amphotericin B with antimonials or paromomycin, improved cure rates and reduced treatment duration but were sometimes associated with increased adverse events [44, 101, 131, 138]. Relapse was most commonly reported among VL patients, particularly those receiving monotherapy, experiencing delayed treatment, or living with immunosuppression, aligning with prior evidence on the chronic and systemic nature of VL [47, 131, 140].

Mucocutaneous and ML required more aggressive treatment regimens, often involving amphotericin B or pentamidine, with generally high cure rates but notable toxicity risks and occasional relapse [42, 137, 143]. Nonulcerated CL demonstrated excellent treatment response with antimonials, although data remain limited [40].

### 4.1. Implications for Policy, Practice, and Research

The findings underscore that leishmaniasis control remains constrained by diagnostic inequities, therapeutic toxicity, and fragile health systems in endemic regions, despite technological advances [8, 9, 144]. The increasing detection of imported cases in nonendemic countries highlights the need for strengthened global surveillance, clinician awareness, and harmonized reporting frameworks [20, 21].

From a research standpoint, major gaps persist in comparative diagnostic accuracy studies, real‐world effectiveness of molecular and point‐of‐care tools, standardized treatment outcome reporting, and long‐term relapse monitoring, particularly for VL, PKDL, and coinfected populations [10–12, 127]. Implementation research is urgently needed to translate diagnostic and therapeutic innovations into routine care in resource‐constrained settings.

The literature reviewed did not identify meaningful incorporation of emerging technologies such as artificial intelligence, machine learning, CRISPR‐based diagnostics, or digital health tools in the clinical diagnosis, treatment, or surveillance of leishmaniasis [151, 154, 155]. Existing studies remain focused on conventional and molecular diagnostics and established therapeutic regimens. The absence is a clear evidence gap, indicating that next‐generation technologies have not yet been translated into routine leishmaniasis research or control programs. Integrating these innovations represents an important future research priority with the potential to enhance diagnostic accuracy, surveillance efficiency, and treatment optimization, particularly in resource‐limited endemic settings.

### 4.2. Strengths and Limitations

This scoping review comprehensively maps the global evidence base across multiple clinical forms, regions, and study designs, offering a broad perspective on leishmaniasis epidemiology, diagnosis, and treatment. However, the absence of formal risk‐of‐bias assessment, restriction to English‐language publications, and heterogeneity in study methodologies limit quantitative comparability and causal inference. Again, preventive strategies were not addressed in this review, as they fall outside the defined scope of the manuscript. The focus of this study was deliberately limited to the geographical distribution, diagnostic approaches, and treatment outcomes of leishmaniasis. Although prevention is critical to disease control, its detailed evaluation warrants a separate, dedicated analysis.

## 5. Conclusion

In conclusion, leishmaniasis remains a highly heterogeneous and persistently neglected disease, characterized by distinct regional patterns, evolving diagnostic technologies, and complex treatment challenges. Although advances in molecular diagnostics and liposomal therapies represent significant progress, equitable access and context‐specific implementation remain critical barriers. Strengthening integrated surveillance, expanding access to accurate diagnostics, optimizing treatment strategies, and addressing key research gaps are essential for reducing the global burden of this enduring NTD.

## Author Contributions

Conceptualization: K.K.A.; data curation: M.W.M., Y.O.A., and S.K.; formal analysis: K.K.A., M.W.M., Y.O.A., and A.A.; methodology: M.W.M., Y.O.A., S.K., and A.A.; writing—original draft: K.K.A.; writing—review and editing: K.K.A., M.W.M., Y.O.A., S.K., and A.A.

## Funding

No funding was received for this manuscript.

## Ethics Statement

The authors have nothing to report.

## Consent

The authors have nothing to report.

## Conflicts of Interest

The authors declare no conflicts of interest.

## Supporting information


**Supporting Information** Additional supporting informa tion can be found online in the Supporting Information section. Table S1: Literature search strategy. Table S2: PRISMA checklist. Table S3: Quality assessment results. Table S4: Joanna Briggs Institute critical appraisal checklist guidelines for quality assessment

## Data Availability

Data sharing is not applicable to this article as no datasets were generated or analyzed during the current study.
