# Supplementary material for: Evidence Gaps and Global Patterns in Leishmaniasis Control: A Scoping Review of Clinical, Diagnostic, and Treatment Strategies
Source: J Parasitol Res. 2026 May 21;2026:8882865. doi: 10.1155/japr/8882865 (PMC13191776; doi:10.1155/japr/8882865)
Supplement: Supplementary file 1 — Supporting Information Additional supporting informa tion can be found online in the Supporting Information section. Table S1: Literature search strategy. Table S2: PRISMA checklist. Table S3: Quality assessment results. Table S4: Joanna Briggs Institute critical appraisal checklist guidelines for quality assessment [file JAPR-2026-8882865-s001.docx]

S1 Table: **Literature Search Strategy**

| **Database** | **Search Formula** | **Number** |
| --- | --- | --- |
| PudMed | (("Leishmaniasis"[MeSH Terms] OR "leishmaniasis, visceral"[MeSH Terms] OR "leishmaniasis, cutaneous"[MeSH Terms] OR "leishmaniasis, mucocutaneous"[MeSH Terms] OR "Leishmaniasis"[Title/Abstract] OR "visceral leishmaniasis"[Title/Abstract] OR "cutaneous leishmaniasis"[Title/Abstract] OR "mucocutaneous leishmaniasis"[Title/Abstract] OR "kala-azar"[Title/Abstract]) AND ("Algeria"[Title/Abstract] OR "Libya"[Title/Abstract] OR "Morocco"[Title/Abstract] OR "Egypt"[Title/Abstract] OR "Eritrea"[Title/Abstract] OR "Kenya"[Title/Abstract] OR "Uganda"[Title/Abstract] OR "Sudan"[Title/Abstract] OR "South Sudan"[Title/Abstract] OR "Somalia"[Title/Abstract] OR "Nigeria"[Title/Abstract] OR "Ghana"[Title/Abstract] OR "Niger"[Title/Abstract] OR "Senegal"[Title/Abstract] OR "Chad"[Title/Abstract] OR "Cameroon"[Title/Abstract] OR "Democratic Republic of the Congo"[Title/Abstract] OR "Zambia"[Title/Abstract] OR "Malawi"[Title/Abstract] OR "Argentina"[Title/Abstract] OR "Belize"[Title/Abstract] OR "Bolivia"[Title/Abstract] OR "Brazil"[Title/Abstract] OR "costa rica"[Title/Abstract] OR "Ecuador"[Title/Abstract] OR "El Salvador"[Title/Abstract] OR "Guyana"[Title/Abstract] OR "Honduras"[Title/Abstract] OR "Mexico"[Title/Abstract] OR "Nicaragua"[Title/Abstract] OR "Panama"[Title/Abstract] OR "Paraguay"[Title/Abstract] OR "Suriname"[Title/Abstract] OR "Venezuela"[Title/Abstract] OR "Afghanistan"[Title/Abstract] OR "Jordan"[Title/Abstract] OR "Kuwait"[Title/Abstract] OR "Lebanon"[Title/Abstract] OR "Oman"[Title/Abstract] OR "Pakistan"[Title/Abstract] OR "Syria"[Title/Abstract] OR "Yemen"[Title/Abstract] OR "Palestinian Territory"[Title/Abstract] OR "India"[Title/Abstract] OR "Bangladesh"[Title/Abstract] OR "Nepal"[Title/Abstract] OR "Spain"[Title/Abstract] OR "Greece"[Title/Abstract] OR "Cyprus"[Title/Abstract] OR "Malta"[Title/Abstract] OR "Albania"[Title/Abstract] OR "Armenia"[Title/Abstract] OR "Azerbaijan"[Title/Abstract] OR "Georgia"[Title/Abstract] OR "Serbia"[Title/Abstract] OR "Montenegro"[Title/Abstract] OR "Bosnia and Herzegovina"[Title/Abstract])) AND ((ffrft[Filter]) AND (humans[Filter]) AND (english[Filter])) | 2,212 |
| Google Scholar | allintitle: ( "leishmaniasis" OR "visceral leishmaniasis" OR "cutaneous leishmaniasis" OR "mucocutaneous leishmaniasis" OR "kala-azar" ) AND ( Algeria OR Libya OR Morocco OR Egypt OR Eritrea OR Kenya OR Uganda OR Sudan OR "South Sudan" OR Somalia OR Nigeria OR Ghana OR Niger OR Senegal OR Chad OR Cameroon OR "Democratic Republic of the Congo" OR Zambia OR Malawi OR Argentina OR Belize OR Bolivia OR Brazil OR "Costa Rica" OR Ecuador OR "El Salvador" OR Guyana OR Honduras OR Mexico OR Nicaragua OR Panama OR Paraguay OR Suriname OR Venezuela OR Afghanistan OR Jordan OR Kuwait OR Lebanon OR Oman OR Pakistan OR Syria OR Yemen OR "Palestinian Territory" OR India OR Bangladesh OR Nepal OR Spain OR Greece OR Cyprus OR Malta OR Albania OR Armenia OR Azerbaijan OR Georgia OR Serbia OR Montenegro OR "Bosnia and Herzegovina" ) leishmaniasis OR "visceral leishmaniasis" OR "cutaneous leishmaniasis" OR "mucocutaneous leishmaniasis" OR "kala azar" OR Algeria OR Libya OR Morocco OR Egypt OR Eritrea OR Kenya OR Uganda OR Sudan OR "South Sudan" OR Somalia OR Nigeria OR Ghana OR Niger OR Senegal OR Chad OR Cameroon OR "Democratic Republic of the Congo" OR Zambia | 648 |
| Science Direct | ("leishmaniasis" OR "visceral leishmaniasis" OR "cutaneous leishmaniasis" OR "mucocutaneous leishmaniasis" OR "kala-azar") AND (Africa OR Asia OR Europe OR Americas) | 1,742 |
| Scopus | (TITLE-ABS-KEY(leishmaniasis in Africa*) AND (TITLE-ABS-KEY(leishmaniasis in Europe*) AND (TITLE-ABS-KEY ( leshmaniasis in Brazil* )AND (TITLE-ABS-KEY ( leshmaniasis in Argentina* ) AND PUBYEAR > 1999AND PUBYEAR < 2027 AND ( LIMIT-TO ( EXACTKEYWORD,"Humans" ) OR LIMIT-TO ( EXACTKEYWORD,"Leishmaniasis" ) ) AND ( LIMIT-TO ( SUBJAREA,"MEDI" ) OR LIMIT-TO ( SUBJAREA,"IMMU" ) OR LIMIT-TO ( SUBJAREA,"BIOC" ) ) AND ( LIMIT-TO ( DOCTYPE,"ar" ) ) AND ( LIMIT-TO ( LANGUAGE,"English" ) ) AND ( LIMIT-TO ( OA,"all" ) ) ) | 785 |
| Total | All Databases | 5,387 |

S2 Table: PRISMA Checklist

| **Section/topic** | **#** | **Checklist item** | **Reported on page #** |
| --- | --- | --- | --- |
| **TITLE** | | |  |
| Title | 1 | Identify the report as a systematic review, meta-analysis, or both. | 1 |
| **ABSTRACT** | | |  |
| Structured summary | 2 | Provide a structured summary including, as applicable: background; objectives; data sources; study eligibility criteria, participants, and interventions; study appraisal and synthesis methods; results; limitations; conclusions and implications of key findings; systematic review registration number. | 1-2 |
| **INTRODUCTION** | | |  |
| Rationale | 3 | Describe the rationale for the review in the context of what is already known. | 3-4 |
| Objectives | 4 | Provide an explicit statement of questions being addressed with reference to participants, interventions, comparisons, outcomes, and study design (PICOS). | 4 |
| **METHODS** | | |  |
| Protocol and registration | 5 | Indicate if a review protocol exists, if and where it can be accessed (e.g., Web address), and, if available, provide registration information including registration number. | 4-5 |
| Eligibility criteria | 6 | Specify study characteristics (e.g., PICOS, length of follow-up) and report characteristics (e.g., years considered, language, publication status) used as criteria for eligibility, giving rationale. | 4-5 |
| Information sources | 7 | Describe all information sources (e.g., databases with dates of coverage, contact with study authors to identify additional studies) in the search and date last searched. | 5 |
| Search | 8 | Present full electronic search strategy for at least one database, including any limits used, such that it could be repeated. | 5 |
| Study selection | 9 | State the process for selecting studies (i.e., screening, eligibility, included in systematic review, and, if applicable, included in the meta-analysis). | 4-5 |
| Data collection process | 10 | Describe method of data extraction from reports (e.g., piloted forms, independently, in duplicate) and any processes for obtaining and confirming data from investigators. | 5 |
| Data items | 11 | List and define all variables for which data were sought (e.g., PICOS, funding sources) and any assumptions and simplifications made. | 5-6 |
| Risk of bias in individual studies | 12 | Describe methods used for assessing risk of bias of individual studies (including specification of whether this was done at the study or outcome level), and how this information is to be used in any data synthesis. | 6 |
| Summary measures | 13 | State the principal summary measures (e.g., risk ratio, difference in means). | - |
| Synthesis of results | 14 | Describe the methods of handling data and combining results of studies, if done, including measures of consistency (e.g., I^2^) for each meta-analysis. | - |

Page 1 of 2

| **Section/topic** | **#** | **Checklist item** | **Reported on page #** |
| --- | --- | --- | --- |
| Risk of bias across studies | 15 | Specify any assessment of risk of bias that may affect the cumulative evidence (e.g., publication bias, selective reporting within studies). | - |
| Additional analyses | 16 | Describe methods of additional analyses (e.g., sensitivity or subgroup analyses, meta-regression), if done, indicating which were pre-specified. | - |
| **RESULTS** | | |  |
| Study selection | 17 | Give numbers of studies screened, assessed for eligibility, and included in the review, with reasons for exclusions at each stage, ideally with a flow diagram. | 4-5 |
| Study characteristics | 18 | For each study, present characteristics for which data were extracted (e.g., study size, PICOS, follow-up period) and provide the citations. | 6-12 |
| Risk of bias within studies | 19 | Present data on risk of bias of each study and, if available, any outcome level assessment (see item 12). | - |
| Results of individual studies | 20 | For all outcomes considered (benefits or harms), present, for each study: (a) simple summary data for each intervention group (b) effect estimates and confidence intervals, ideally with a forest plot. | - |
| Synthesis of results | 21 | Present results of each meta-analysis done, including confidence intervals and measures of consistency. | - |
| Risk of bias across studies | 22 | Present results of any assessment of risk of bias across studies (see Item 15). | - |
| Additional analysis | 23 | Give results of additional analyses, if done (e.g., sensitivity or subgroup analyses, meta-regression [see Item 16]). |  |
| **DISCUSSION** | | |  |
| Summary of evidence | 24 | Summarize the main findings including the strength of evidence for each main outcome; consider their relevance to key groups (e.g., healthcare providers, users, and policy makers). | 12-16 |
| Limitations | 25 | Discuss limitations at study and outcome level (e.g., risk of bias), and at review-level (e.g., incomplete retrieval of identified research, reporting bias). | 15 |
| Conclusions | 26 | Provide a general interpretation of the results in the context of other evidence, and implications for future research. | 15 |
| **FUNDING** | | |  |
| Funding | 27 | Describe sources of funding for the systematic review and other support (e.g., supply of data); role of funders for the systematic review. | - |

S3 Table: **Quality assessment results**

|  |  | **Quality criteria score** | | | | | | | | |  |
| --- | --- | --- | --- | --- | --- | --- | --- | --- | --- | --- | --- |
| **Study** | **country** | **A** | **B** | **C** | **D** | **E** | **F** | **G** | **H** | **I** | **Total** |
| Dabirzadeh et al. [27] | Iran | 1 | 1 | 1 | 1 | 1 | 1 | 1 | 1 | 1 | 9 |
| Abukhattab et al. [28] | Qatar | 1 | 1 | 1 | 1 | 1 | 1 | 1 | 1 | 1 | 9 |
| Alharbi et al. [29] | Saudi Arabia | 1 | 1 | 1 | 1 | 0 | 1 | 1 | 1 | 1 | 8 |
| Yizengaw et al. [30] | Ethiopia | 1 | 1 | 1 | 1 | 1 | 1 | 1 | 1 | 1 | 9 |
| Lindner et al. [31] | Germany | 1 | 1 | 1 | 1 | 1 | 1 | 1 | 1 | 1 | 9 |
| Rocha et al. [32] | Portugal | 1 | 1 | 1 | 1 | 1 | 1 | 1 | 1 | 1 | 9 |
| Debash et al. [33] | Ethiopia | 1 | 1 | 0 | 1 | 1 | 1 | 1 | 0 | 1 | 7 |
| Alzahrani et al. [34] | Saudi Arabia | 1 | 1 | 1 | 1 | 1 | 1 | 1 | 1 | 1 | 9 |
| Aflatoonian et al. [35] | Iran | 1 | 1 | 1 | 1 | 1 | 1 | 1 | 1 | 1 | 9 |
| Abdullahi et al. [36] | Kenya | 1 | 1 |  | 1 | 1 |  | 1 | 1 | 1 | 8 |
| Aissaoui et al. [37] | France | 1 | 1 | 1 | 1 | 1 | 1 | 1 | 1 | 1 | 9 |
| Aalto et al. [38] | Somalia | 1 | 1 | 1 | 1 | 1 | 1 | 1 | 1 | 1 | 9 |
| Alhawarat et al. [39] | Jordan | 0 | 1 | 1 | 1 | 0 | 1 | 0 | 1 | 1 | 7 |
| Kanyina et al. [40] | Kenya | 1 | 1 | 1 | 1 | 1 | 1 | 1 | 1 | 1 | 9 |
| Bisetegn et al. [41] | Ethiopia | 1 | 1 | 1 | 1 | 1 | 1 | 1 | 1 | 1 | 9 |
| Baghad et al. [42] | Morocco | 1 | 1 | 1 | 1 | 1 | 1 | 1 | 1 | 1 | 9 |
| Abou-Elaaz et al. [43] | Morocco | 1 | 1 | 1 | 1 | 1 | 1 | 1 | 1 | 1 | 9 |
| Hawash et al. [44] | Saudi Arabia | 1 | 1 | 1 | 1 | 1 | 1 | 1 | 1 | 1 | 9 |
| Mokhtar et al. [45] | Saudi Arabia | 1 | 1 | 1 | 1 | 1 | 1 | 1 | 1 | 1 | 9 |
| Castro et al. [46] | Colombia | 1 | 1 | 1 | 1 | 1 | 1 | 1 | 1 | 1 | 9 |
| Elmekki et al. [47] | Saudi Arabia | 1 | 1 | 1 | 1 | 1 | 1 | 1 | 1 | 1 | 9 |
| Helel et al. [48] | Tunisia | 1 | 1 | 1 | 1 | 1 | 1 | 1 | 1 | 1 | 9 |
| Traore et al. [49] | Mali | 1 | 1 | 1 | 1 | 0 | 1 | 1 | 1 | 1 | 8 |
| EL Aasri et al. [50] | Morocco | 1 | 1 | 1 | 1 | 1 | 1 | 1 | 1 | 1 | 9 |
| Abdinia et al. [51] | Iran | 1 | 1 | 1 | 1 | 1 | 1 | 1 | 1 | 1 | 9 |
| Araujo et al. [52] | Brazil | 1 | 1 | 1 | 1 | 1 | 1 | 1 | 1 | 1 | 9 |
| Vita et al. [53] | Brazil | 1 | 1 | 0 | 1 | 1 | 1 | 1 | 0 | 1 | 7 |
| Oré et al. [54] | Peru | 1 | 1 | 1 | 1 | 1 | 1 | 1 | 1 | 1 | 9 |
| Calderaro et al. [55] | Italy | 1 | 1 | 1 | 1 | 1 | 1 | 1 | 1 | 1 | 9 |
| Yemisen et al. [56] | Turkey | 1 | 1 |  | 1 | 1 |  | 1 | 1 | 1 | 8 |
| Oliveira et al. [57] | Mali | 1 | 1 | 1 | 1 | 1 | 1 | 1 | 1 | 1 | 9 |
| AlSamarai et al. [58] | Iraq | 1 | 1 | 1 | 1 | 1 | 1 | 1 | 1 | 1 | 9 |
| Negera et al. [59] | Ethiopia | 0 | 1 | 1 | 1 | 0 | 1 | 0 | 1 | 1 | 7 |
| Vieira-Gonçalves et al. [60] | Brazil | 1 | 1 | 1 | 1 | 1 | 1 | 1 | 1 | 1 | 9 |
| Lawn et al. [61] | UK | 1 | 1 | 1 | 1 | 1 | 1 | 1 | 1 | 1 | 9 |
| Al-Qurashi et al. [62] | Saudi Arabia | 1 | 1 | 1 | 1 | 1 | 1 | 1 | 1 | 1 | 9 |
| Israël et al. [63] | Chad | 1 | 1 | 1 | 1 | 1 | 1 | 1 | 1 | 1 | 9 |
| Vutova et al. [64] | Bulgaria | 1 | 1 | 1 | 1 | 1 | 1 | 1 | 1 | 1 | 9 |
| Saadene et al. [65] | Algeria | 1 | 1 | 1 | 1 | 1 | 1 | 1 | 1 | 1 | 9 |
| Almazán et al. [66] | Argentina | 1 | 1 | 1 | 1 | 1 | 1 | 1 | 1 | 1 | 9 |
| Chowdhury et al. [67] | Bangladesh | 1 | 1 | 1 | 1 | 1 | 1 | 1 | 1 | 1 | 9 |
| Zhao et al. [68] | China | 1 | 1 | 1 | 1 | 1 | 1 | 1 | 1 | 1 | 9 |
| Kuhls et al. [69] | Armenia | 1 | 1 | 1 | 1 | 0 | 1 | 1 | 1 | 1 | 8 |
| Ballart et al. [70] | Bolivia | 1 | 1 | 1 | 1 | 1 | 1 | 1 | 1 | 1 | 9 |
| Adegboye & Adegboye [71] | Afghanistan | 1 | 1 | 1 | 1 | 1 | 1 | 1 | 1 | 1 | 9 |
| Diadie et al. [72] | Senegal | 1 | 1 | 1 | 1 | 1 | 1 | 1 | 1 | 1 | 9 |
| Garrido-Jareño et al. [73] | Spain | 1 | 1 | 0 | 1 | 1 | 1 | 1 | 0 | 1 | 7 |
| Galgamuwa et al [74] | Sri lanka | 1 | 1 | 1 | 1 | 1 | 1 | 1 | 1 | 1 | 9 |
| Iddawela et al. [75] | Sri Lanka | 1 | 1 | 1 | 1 | 1 | 1 | 1 | 1 | 1 | 9 |
| Díaz-Sáez et al. [76] | Spain | 1 | 1 |  | 1 | 1 |  | 1 | 1 | 1 | 8 |
| Schallig et al. [77] | Suriname | 1 | 1 | 1 | 1 | 1 | 1 | 1 | 1 | 1 | 9 |
| Larréché et al.[78] | Turkmenistan | 1 | 1 | 1 | 1 | 1 | 1 | 1 | 1 | 1 | 9 |
| Zhao et al. [79] | China | 0 | 1 | 1 | 1 | 0 | 1 | 0 | 1 | 1 | 7 |
| Bi et al.[80] | Venezuela | 1 | 1 | 1 | 1 | 1 | 1 | 1 | 1 | 1 | 9 |
| De Lima et al. [81] | Thailand | 1 | 1 | 1 | 1 | 1 | 1 | 1 | 1 | 1 | 9 |
| Jundang et al. [82] | Palestine | 1 | 1 | 1 | 1 | 1 | 1 | 1 | 1 | 1 | 9 |
| Ahmad et al. [83] | USA | 1 | 1 | 1 | 1 | 1 | 1 | 1 | 1 | 1 | 9 |
| Pinart et al. [84] | Motenegro | 1 | 1 | 1 | 1 | 1 | 1 | 1 | 1 | 1 | 9 |
| Medenica et al. [85] | Pakistan | 1 | 1 | 1 | 1 | 1 | 1 | 1 | 1 | 1 | 9 |
| Iqbal et al.[86] | Kuwait | 1 | 1 | 1 | 1 | 1 | 1 | 1 | 1 | 1 | 9 |
| Iqbal et al. [87] | Malta | 1 | 1 | 1 | 1 | 1 | 1 | 1 | 1 | 1 | 9 |
| Grech et al. [88] | Panama | 1 | 1 | 1 | 1 | 1 | 1 | 1 | 1 | 1 | 9 |
| Gonzalez et al. [89] | Mauritania | 1 | 1 | 1 | 1 | 0 | 1 | 1 | 1 | 1 | 8 |
| El Moctar et al. [90] | Lebanon | 1 | 1 | 1 | 1 | 1 | 1 | 1 | 1 | 1 | 9 |
| El Hajj et al.[91] | Niger | 1 | 1 | 1 | 1 | 1 | 1 | 1 | 1 | 1 | 9 |
| Blaizot et al. [92] | Libya | 1 | 1 | 1 | 1 | 1 | 1 | 1 | 1 | 1 | 9 |
| Amro et al. [93] | Isreal | 1 | 1 | 0 | 1 | 1 | 1 | 1 | 0 | 1 | 7 |
| Solomon et al. [94] | Oti Region (Ghana) | 1 | 1 | 1 | 1 | 1 | 1 | 1 | 1 | 1 | 9 |
| Akuffo et al. [95] | Bihar (Vaishali) | 1 | 1 | 1 | 1 | 1 | 1 | 1 | 1 | 1 | 9 |
| Kumar et al. [96] | India | 1 | 1 |  | 1 | 1 |  | 1 | 1 | 1 | 8 |
| Kato et al. [97] | Georgia (Kakheti) | 1 | 1 | 1 | 1 | 1 | 1 | 1 | 1 | 1 | 9 |
| Babuadze et al. [98] | Georgia (Kakheti) | 1 | 1 | 1 | 1 | 1 | 1 | 1 | 1 | 1 | 9 |
| Kimutai et al. [99] | Guinea/ West Africa | 0 | 1 | 1 | 1 | 0 | 1 | 0 | 1 | 1 | 7 |
| Sosa-Ochoa et al. [100] | Honduras (Amapala) | 1 | 1 | 1 | 1 | 1 | 1 | 1 | 1 | 1 | 9 |
| Thakur et al. [101] | India (Himachal Pradesh) | 1 | 1 | 1 | 0 | 1 | 1 | 1 | 1 | 1 | 8 |

**Definition of terms**: **A**; appropriateness of sample frame to address the target population, **B**; appropriateness of the way used to sample study participants, **C**; adequateness of sample size, **D**; description of study subjects and settings, **E**; data analysis coverage in the identified sample, **F**; validity of method used to identify schistosomiasis reinfection rate, **G**; reliability of method used to measure schistosomiasis reinfection rate for all participants, **H**; appropriateness of statistical tests used in data analysis and **I**; adequateness of response rate

S4. Table: Joanna Briggs Institute critical appraisal checklist guidelines for Quality assessment

| **Term** | **Definition** |
| --- | --- |
| Critical appraisal | The process of systematically assessing the outcome of scientific research to judge its trustworthiness, value, and relevance in a particular context. |
| External validity | Applicability of the findings to a given population. |
| Generalizability | The degree to which the results of a study can be applied to a broader population or situation. |
| Imprecision | The GRADE approach to rating imprecision focuses on the 95% CI around the best estimate of the absolute effect. |
| Indirectness | The GRADE approach to rating indirectness focuses on concerns about how the population, intervention, or outcomes differ from those of interest. |
| Internal validity | A measure of how well a study is conducted and how accurately its results reflect the studied group. |
| Methodological quality | The extent to which there is potential for errors and bias in the design and execution of a study. |
| Power | The probability of finding a statistically significant result. |
| Publication bias | The likelihood studies have not been published based on the outcome of the research study. |
| Random error | An error in measurement caused by factors that vary from one measurement to another. |
| Reporting quality | The extent to which a complete and transparent description of the design, conduct, and analysis of a study is given. |
| Risk of bias | The likelihood that features of the study design or conduct of the study will give misleading results. |
| Sample size | The number of participants or observations included in a study. |
| Statistical conclusion validity | The extent to which the conclusions of research are founded on adequate analysis of the data. |
| Systematic error | Errors that affect the accuracy of a measurement or cause readings to differ from the true value by a consistent amount each time a measurement is made. |
